# Engineered biomimetic nanoparticles achieve targeted delivery and efficient metabolism-based synergistic therapy against glioblastoma

**DOI:** 10.1038/s41467-022-31799-y

**Published:** 2022-07-21

**Authors:** Guihong Lu, Xiaojun Wang, Feng Li, Shuang Wang, Jiawei Zhao, Jinyi Wang, Jing Liu, Chengliang Lyu, Peng Ye, Hui Tan, Weiping Li, Guanghui Ma, Wei Wei

**Affiliations:** 1 Department of Neurosurgery, Health Science Center, The First Affiliated Hospital of Shenzhen University, Shenzhen Second People’s Hospital, Shenzhen, 518035 P. R. China; 2 State Key Laboratory of Biochemical Engineering, Institute of Process Engineering, Chinese Academy of Sciences, Beijing, 100190 P. R. China; 3 School of Chemical Engineering, University of Chinese Academy of Sciences, Beijing, 100049 P. R. China; 4 Pneumology Department, Shenzhen Children’s Hospital, Shenzhen, 518026 P. R. China

**Keywords:** Drug delivery, Targeted therapies, CNS cancer

## Abstract

Glioblastoma multiforme (GBM) is an aggressive brain cancer with a poor prognosis and few treatment options. Here, building on the observation of elevated lactate (LA) in resected GBM, we develop biomimetic therapeutic nanoparticles (NPs) that deliver agents for LA metabolism-based synergistic therapy. Because our self-assembling NPs are encapsulated in membranes derived from glioma cells, they readily penetrate the blood-brain barrier and target GBM through homotypic recognition. After reaching the tumors, lactate oxidase in the NPs converts LA into pyruvic acid (PA) and hydrogen peroxide (H_2_O_2_). The PA inhibits cancer cell growth by blocking histones expression and inducing cell-cycle arrest. In parallel, the H_2_O_2_ reacts with the delivered bis[2,4,5-trichloro-6-(pentyloxycarbonyl)phenyl] oxalate to release energy, which is used by the co-delivered photosensitizer chlorin e6 for the generation of cytotoxic singlet oxygen to kill glioma cells. Such a synergism ensures strong therapeutic effects against both glioma cell-line derived and patient-derived xenograft models.

## Abstract (web summary)

Targeting cancer-associated metabolism is evolving as a promising approach for cancer therapy. Here, the authors generate cancer cell-membrane encapsulated nanoparticles to induce cell cycle arrest and cytotoxicity in lactate-high cancer cells, reducing tumourigensis in glioblastoma cell-line and patient-derived models.

## Introduction

Glioblastoma multiforme (GBM) is one of the most aggressive intracranial tumors, and remains clinically incurable^1,2^. So far, the standard treatment for GBM is surgery followed by radiotherapy with concurrent chemotherapy^3–5^. Because of its particular location in the human body and the aggressive characteristics of glioblastomas, surgery alone is insufficient to eradicate the pathological tissue completely. The current radiotherapy approach results low response rate and severe toxicity, thus limiting its application. Chemotherapy, which is another therapy method used for GBM, often causes serious side effects, and its efficacy is severely limited by poor drugs penetration due to the blood–brain barrier (BBB). Despite advances made in surgery, radiotherapy, and chemotherapy in the past years, GBM patients still have a median survival of only about 14 months and a 5-year survival rate of 4–5% after the first diagnosis^6^. Thus, effective therapeutic modalities for treating GBM are urgently needed.

Altered cellular metabolism is a hallmark of cancer, including GBM^7,8^. Compared to normal tissue, the metabolic activity in the tumors is reprogrammed for almost every major metabolic pathway, including glycolysis, the tricarboxylic acid (TCA) cycle, the pentose phosphate pathway, and amino acid metabolism^9,10^. Among these, glycolysis is regarded as the central pathway in cancer cell metabolism^11^. Through glycolysis, tumor cells consume large amounts of glucose and convert it into lactate (LA), accompanied high level of energy/ATP generation to support rapid proliferation. The accumulated LA, which once was considered as a waste product of glycolysis in the tumor microenvironment (TME), has been proven to play an important role in promoting cancer invasion and metastasis^12–14^. Therefore, aiming LA metabolism is an attractive tumor therapeutic strategy^15,16^. Recent studies have shown that interfering with LA metabolism using small molecular inhibitors and small interfering RNA (siRNA) exhibited potent antitumor effects^17–20^. Despite this progress in LA metabolic therapies, there are no reports that directly harness LA metabolism for GBM treatments. One of the strongest limitations in treating brain tumors is the existence of the BBB, which prevents nearly 98% of small-molecule drugs and most macromolecular drugs (including those interfering with LA metabolism) from reaching the brain^21,22^. Moreover, considering the complexity and infiltrating characteristics of GBM, LA metabolic monotherapy is very likely to be insufficient for the effective elimination of GBM cells^23,24^. Therefore, it is vital to develop synergistic strategies to enhance the therapeutic efficiency of LA metabolic therapy.

The genesis of the present study was our observation of elevated intratumoral LA metabolism in resected tumors from glioma patients. After reconfirming this elevation in a mouse glioma model, we were interested in using LA metabolic therapy as part of the therapeutic strategy against GBM. Moreover, given that LA can be selectively converted into pyruvic acid (PA) and hydrogen peroxide (H_2_O_2_) by lactate oxidase (LOX)^25,26^, we envisioned a system for utilizing the elevated H_2_O_2_ to support a form of chemiexcited photodynamic therapy (chemiexcited PDT)^27–29^. That is, we aimed to deliver LOX to harness LA as a source of local fuel to increase H_2_O_2_, which could subsequently react with a delivered chemiluminescence reagent (e.g., bis[2,4,5-trichloro-6-(pentyloxycarbonyl)phenyl] oxalate, CPPO). The ensuing H_2_O_2_-CPPO reaction would release energy to activate a delivered photosensitizer chlorin e6 (Ce6) to generate cytotoxic singlet oxygen (^1^O_2_) to kill glioma cells.

**Fig. 1:**
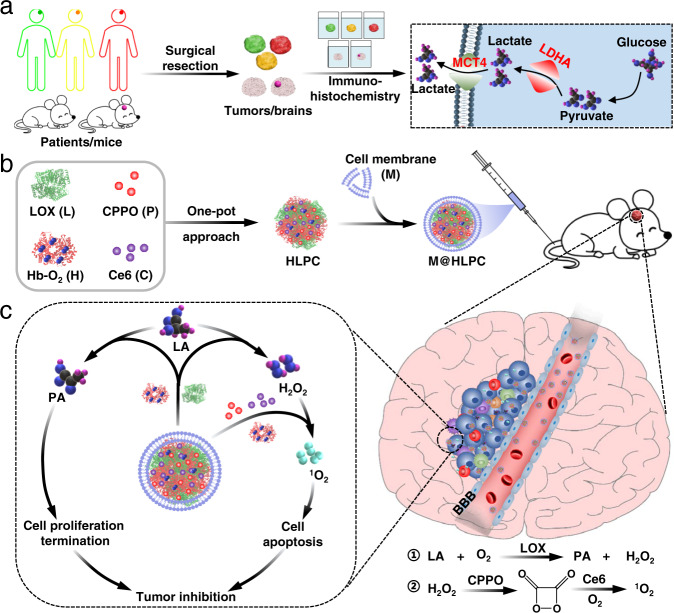
Schematic illustration of clinical and mice glioma analysis and M@HLPC construction for tumor inhibition. **a** Analysis of lactate (LA) metabolism-associated indicators (lactate dehydrogenase A, LDHA; monocarboxylate transporter 4, MCT4) in tumor samples from patients and mice gliomas. **b** Schematic illustration of the construction of M@HLPC. The self-assembly NPs composed of oxygen-carrying Hb-O_2_, LOX, CPPO, and Ce6 (denoted as HLPC) are fabricated using a one-pot approach, followed by coating with the M prepared from glioma cells. **c** Targeted accumulation of M@HLPC in GBM and subsequent synergistic therapy upon the combination of LA metabolic therapy and chemiexcited PDT. M@HLPC are able to cross the BBB and actively target GBM due to the homotypic targeting ability of glioma cell membranes. After accumulation in the glioma site, the LOX in M@HLPC can selectively convert the intra-tumor LA into PA and H_2_O_2_ upon consumption of O_2_ carried by Hb (①). Furthermore, the in situ-produced PA can act as a signaling molecule to repress histone gene expression, delay cell cycle progression, and terminate cell proliferation. At the same time, the released H_2_O_2_ can react with CPPO to produce chemical energy, which can activate the photosensitizer Ce6 to generate ^1^O_2_ and induce cell apoptosis (②), achieving chemiexcited PDT without laser irradiation. This design enables M@HLPC to achieve targeted accumulation in GBM and then support both LA metabolic therapy and chemiexcited PDT in a synergistic manner.

This report details our development, proof-of-concept demonstration, and pre-clinical testing of a tumor cell membrane-coated biomimetic nanoparticles (NPs) system that can cross the BBB and actively home to GBM cells (based on homotypic recognition of membrane components), where it releases the agents needed for combined LA metabolic therapy and chemiexcited PDT (Fig. 1). Very briefly, the NPs were obtained via the self-assembly of hemoglobin (Hb, as a source of molecular oxygen for both LA metabolic therapy and chemiexcited PDT)^30^, LOX (to convert LA into PA and H_2_O_2_), and CPPO-Ce6 (for chemiexcited PDT). We subsequently encapsulated these self-assembled NPs with membrane materials (M) prepared from U251 glioma cells to generate the biomimetic M@HLPC system. After confirming the respective functionality of each M@HLPC sub-component, we observed strong antitumor performance of our biomimetic NPs based on synergistic LA metabolic therapy and chemiexcited PDT in both cell-line derived xenograft (CDX) and patient-derived xenograft (PDX) tumor models.

## Results

### High expression of representative LA production and efflux indicators in glioma patients and glioma-bearing mice

**Fig. 2:**
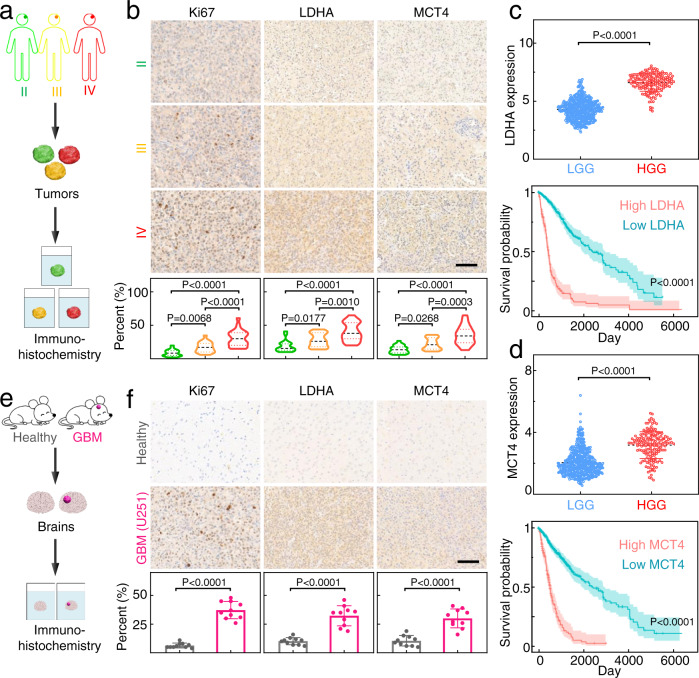
Profiling of LA metabolism-associated indicators expression in gliomas obtained from patients or mice. **a** Schematic illustration of LA metabolism-related indicators (LDHA and MCT4) analysis in tumor samples from glioma patients. **b** IHC images and quantitative analysis of the expression of Ki67, LDHA, and MCT4 in grade II, III, and IV gliomas resected from glioma patients (II: *n* = 22, III: *n* = 21, IV: *n* = 23). Scale bar: 100 μm. **c** Assessment of LDHA expression and the corresponding survival probability of glioma patients. Data from The Cancer Genome Atlas (TCGA) database, including 524 low-grade glioma (LGG) and 167 high-grade glioma (HGG) cases. Each dot represents an individual case. The OncoLnc tool was used to explore the relationship between survival probability and LDHA levels. **d** MCT4 expression and the corresponding survival probability of the same glioma samples in panel (**c**). The OncoLnc tool was used to explore the relationship between survival probability and MCT4 levels. **e** Schematic illustration of LA metabolism-related indicators analysis for brains from healthy and U251 CDX tumor-bearing mice. **f** IHC images and quantitative analysis for the expression of Ki67, LDHA, and MCT4 in brain samples from healthy mice and U251 CDX tumor-bearing mice (*n* = 1 experiment, *n* = 10 mice per group). Scale bar: 100 μm. Data in (**b**–**d**, **f**) were presented as the mean ± SD. *P* values were calculated by using one-way ANOVA (**b**) or two-tailed unpaired Student’s *t*-test (**c**, **d**, **f**). Source data are provided in the Source data file.

Given reports that high LA levels were associated with tumor progression, metastases, recurrence, and reduced patient survival^12,31^, we used immunohistochemistry (IHC) to measure the expression of representative proliferation marker Ki67, LA production indicator lactate dehydrogenase A (LDHA), and LA efflux indicator monocarboxylate transporter 4 (MCT4) in glioma samples from patients (Fig. 2a). We detected a clear trend: the Ki67, LDHA, and MCT4 levels were increased from grade II to grade III and gained to grade IV (Fig. 2b), and there was a positive correlation between the levels of above LA metabolic indicators and the extent of glioma proliferation (Supplementary Fig. 1). We also assessed LDHA and MCT4 levels in glioma samples from The Cancer Genome Atlas (TCGA), including 524 cases of low-grade gliomas (LGG) and 167 cases of high-grade gliomas (HGG). The HGG samples had significantly higher (*p* < 0.0001) levels of LDHA and MCT4 than the LGG samples did (Fig. 2c, d, upper panel). The Kaplan–Meier survival curve analysis of the association between LDHA/MCT4 and mortality indicated that glioma patients with lower LDHA and MCT4 expression had prolonged survival time (Fig. 2c, b, lower panel). These results indicated that LA levels were significantly elevated in HGG and played an important role in glioma development.

Extending these findings from clinical data to animal models, our IHC analysis of the brains of healthy and U251 tumor-bearing mice showed that the LDHA and MCT4 levels were significantly higher in the glioma brains than in normal brains (Fig. 2e, f). Abovementioned clinical findings and confirmatory animal model results motivated us to harness the elevated LA in GBM to develop an “LA metabolic therapy” against GBM. Recalling that LA can be selectively converted into PA and H_2_O_2_ by LOX and PA is known to function as a signaling molecule for repressing histone gene expression and inducing cell-cycle arrest^32^, we thus proposed to utilize LOX to terminate cell proliferation for metabolic therapy. Further, we were interested in the idea of using the produced H_2_O_2_ as a reactant in a chemical reaction with CPPO to produce ^1^O_2_ for inducing cell apoptosis, potentially enabling the so-called chemiexcited PDT^27–29^.

### Construction and characterizations of M@HLPC

**Fig. 3:**
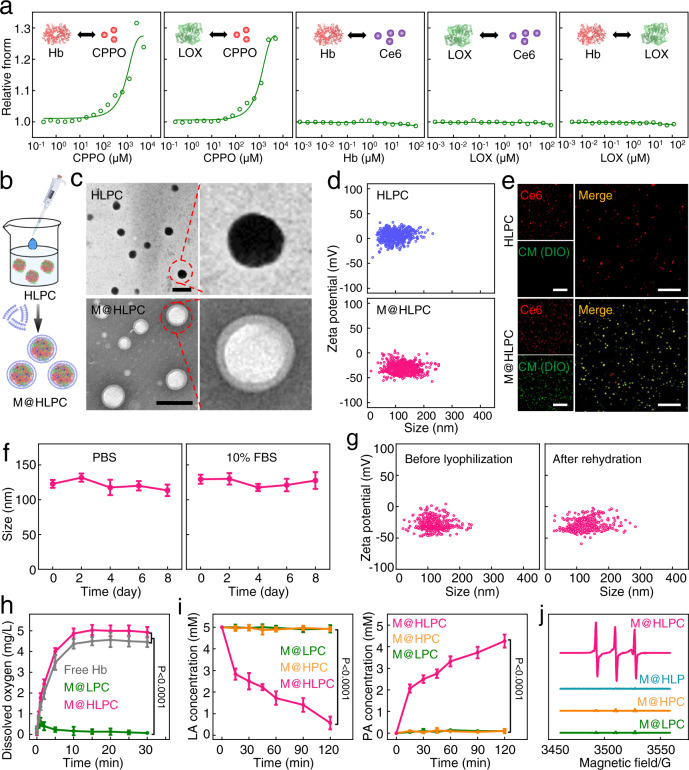
Construction and characterization of M@HLPC. **a** Fitted sigmoidal binding curve of temperature-jump microscale thermophoresis (MST) signal for Hb-CPPO, LOX-CPPO, Hb-Ce6, LOX-Ce6, and Hb-LOX interactions. **b** Schematic illustration of the preparation of HLPC and M@HLPC. **c** TEM images of HLPC and M@HLPC. M@HLPC was negatively stained with 2% phosphotungstic acid solution before imaging. Scale bar: 200 nm. **d** Size and zeta potential distribution of HLPC and M@HLPC analyzed using nanoparticle tracking analysis (NTA). **e** Confocal laser scanning microscopy (CLSM) images of HLPC and M@HLPC. Note that the Ce6 (inside the formed HLPC) is known to emit strong fluorescence (false colored in red here); the green signal was from DIO, which was used to label the coated M. Scale bar: 25 μm. **f** Stability of M@HLPC over the 8-day period in PBS or PBS with 10% FBS. **g** Particle size and zeta potential of M@HLPC before and after lyophilization/rehydration. **h** Oxygen release profiles of free Hb, M@LPC (lacking Hb-O_2_), and M@HLPC in hypoxic PBS, analyzed using a portable oxygen meter. **i** Reaction evolution of LA (5 mM) oxidation by M@LPC (lacking Hb-O_2_), M@HPC (lacking LOX), and M@HLPC, analyzed with Lactate and Pyruvate Assay Kit in hypoxic PBS. **j** ESR spectroscopy of M@LPC (lacking Hb-O_2_), M@HPC (lacking LOX), M@HLP (lacking Ce6), and M@HLPC in hypoxic PBS for analysis of ^1^O_2_ signal intensity upon addition of 5 mM LA. Data in (**f**), (**h**), (**i**) were presented as the mean ± SD, *n* = 3 independent samples. The experiments in (**c**–**e**, **g**) were repeated independently three times with similar results. *P* values were calculated by using one-way ANOVA. Source data are provided in the Source data file.

The abovementioned results prompted us to build a delivery system to synergize LA metabolic therapy with chemiexcited PDT. For this purpose, we fabricated the self-assembly NPs composed of Hb, LOX, CPPO, and Ce6 (denoted as HLPC) using a one-pot approach based on the strong interaction between CPPO and proteins (Hb and LOX) (Fig. 3a). After optimization (Supplementary Fig. 2), the contents of Hb, LOX, CPPO, and Ce6 were determined to be 78.8 ± 0.2, 6.7 ± 0.9, 9.4 ± 0.7, and 5.1 ± 0.4 µg per 100 µg NPs, respectively (Supplementary Fig. 3). Subsequently, the fabricated HLPC and membrane materials (M) harvested from U251 cells were coextruded through a porous membrane to generate M coated HLPC (denoted M@HLPC) (Fig. 3b). The M camouflage was observed in transmission electron microscopy (TEM) images, where the HLPC were surrounded by a membrane layer (Fig. 3c). In addition, the increased hydrodynamic diameter (from 98.3 nm in HLPC to 118.7 nm in M@HLPC) and altered surface charge (from 10.5 mV in HLPC to −29.1 mV in M@HLPC) (Fig. 3d), accompanied with the perfect colocalization of HLPC signal (red fluorescence; from Ce6) and M (green fluorescence; from 3,3′-dioctadecyloxacarbocyanine perchlorate (DiO)-labeled M) (Fig. 3e), further confirmed the successful coating of M on the HLPC. Moreover, M@HLPC demonstrated a plausible feature of stability, with no detectable changes in particle size or zeta potential after storage in phosphate-buffered saline (PBS) or PBS with 10% fetal bovine serum (FBS) medium at 4 °C (Fig. 3f), even after lyophilization and rehydration (Fig. 3g).

Having successfully prepared M@HLPC, we further assessed whether every component comprising M@HLPC retained its desired functionality. As shown in Fig. 3h, compared with the negligibly dissolved oxygen (O_2_) from M@LPC (lacking Hb-O_2_), gradually released O_2_ was detected in M@HLPC and free Hb sample, demonstrating that the Hb in M@HLPC retained the ability of carrying and releasing O_2_ in the hypoxia environment. Furthermore, lactate oxidation activity conversion in hypoxia condition showed that M@LPC (lacking Hb-O_2_) and M@HPC (lacking LOX) could not catalyze LA into PA (Fig. 3i). In contrast, near stoichiometric conversion of LA into PA was found in M@HLPC group, which indicating that the LOX in M@HLPC could effectively convert LA into PA upon consuming O_2_ carried by Hb. In addition, the absorption properties of Ce6 were well maintained, and the stability of Ce6 was improved after the efficient incorporation (Supplementary Fig. 4), which ensured the potential activity of Ce6 for ^1^O_2_ generation during the chemiexcited PDT.

Recalling from our design that LOX-catalyzed PA production was accompanied by the release of H_2_O_2_, which could react with the CPPO component to produce chemical energy for the photosensitizer Ce6 to generate ^1^O_2_ as a cytotoxic agent, we thus tested the ^1^O_2_ generation capacity in four differently-fabricated NPs, including M@LPC (lacking Hb-O_2_), M@HPC (lacking LOX), M@HLP (lacking Ce6), and M@HLPC in hypoxic PBS. As shown in Fig. 3j, there were almost no characteristic ^1^O_2_ peaks in the electron spin resonance (ESR) spectra of M@LPC, M@HPC, and M@HLP dissolved in LA-rich solution. In contrast, the appearance of the three characteristic peaks in the ESR spectra demonstrated ^1^O_2_ generation from M@HLPC in the same conditions. Fluorescence analysis using the previously reported 9,10-Anthracenediyl-bis(methylene) dimalonic acid (ABDA) probe^33^ also clearly showed that only M@HLPC samples exhibited decreased ABDA signals, and this signal decrease was LA concentration-dependent (Supplementary Fig. 5). These results collectively supported the assertion that the therapeutic functions of our M@HLPC system worked as designed: M@HLPC responded to LA, generated PA for potential metabolic therapy, and released H_2_O_2_ for ^1^O_2_ generation through the CPPO-excited Ce6 (without any external excitation light).

### BBB penetration and glioma-targeting performance

**Fig. 4:**
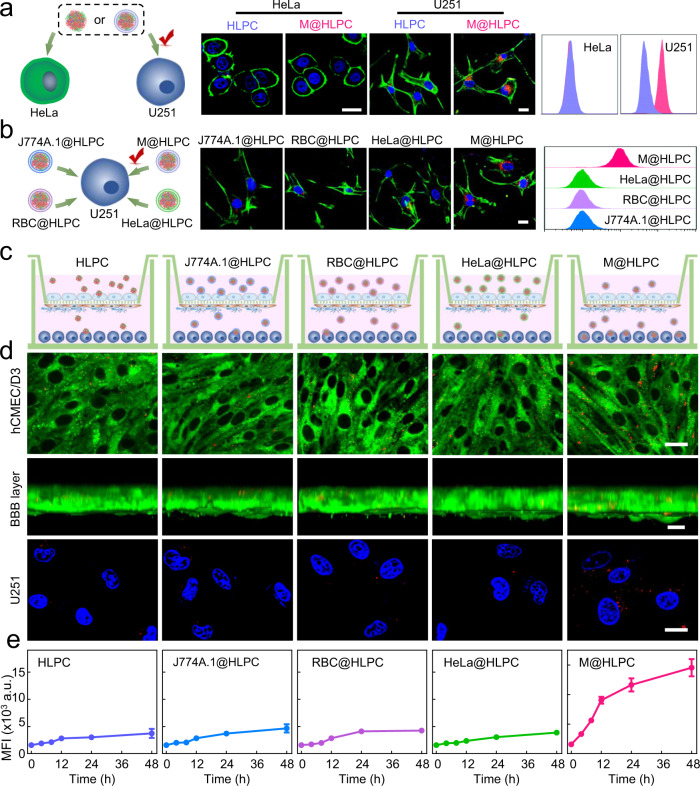
In vitro homotypic tumor cell uptake and BBB penetration ability of M@HLPC. **a** Schematic illustration, representative CLSM images, and corresponding flow cytometry analysis in the experiment testing homotypic tumor cell uptake of nanoparticles (NPs). Human cervical carcinoma cells (HeLa) or human glioma cells (U251, the source of M used in the coating for the M@HLPC) were incubated with the same concentrations of bare HLPC or M@HLPC. Red: NPs (Ce6 signal); blue: Hoechst 33342-labeled nuclei; green: Rhodamine phalloidin-labeled HeLa or U251 cells. Scale bar: 25 μm. **b** Schematic illustration, representative CLSM images, and corresponding flow cytometry analysis of an experiment examining the influence of the M source on the glioma cell homing ability. HLPC were coated with M harvested from the diverse cell lines (macrophage J774A.1, red blood cells (RBC), HeLa, U251), and then incubated with U251 cells. Red: NPs (Ce6 signal); blue: Hoechst 33342-labeled nuclei; green: Rhodamine phalloidin-labeled U251 cells. Scale bar: 25 μm. **c** Schematic illustration of the in vitro BBB model (Transwell™) for evaluating the potential BBB penetration ability of the bare HLPC and those coated with diverse M. **d** Representative CLSM images of the hCMEC/D3 monolayer, BBB layer, and U251 cells showed the penetration and targeting ability of various NPs. Red: NPs; blue: Hoechst 33342-labeled nuclei; green: DIO-labeled BBB layer cell membrane. Scale bar: 25 μm. **e** Quantification analysis of time-dependent internalization of NPs by U251 cells. Data in e were presented as the mean ± SD, *n* = 3 independent samples. The experiments in (**a**, **b**, **d**) were repeated independently three times with similar results. Source data are provided in the Source data file.

After demonstrating the functionality of each M@HLPC sub-component, we next attempted to estimate the M@HLPC uptake by the parent cells^34^. To this end, human cervical carcinoma cells (HeLa) or human glioma cells (U251, the source of the M used in the coating for the M@HLPC) were treated with HLPC or M@HLPC, and the uptake behaviors were analyzed using confocal laser scanning microscopy (CLSM) and flow cytometry. As shown in Fig. 4a and Supplementary Fig. 6, both HLPC and M@HLPC failed to induce effective uptake by HeLa. In contrast, M@HLPC induced a much higher uptake efficiency in U251 cells than the bare HLPC did, revealing a U251-specific uptake preference for M@HLPC. For further verification, U251 cells were separately treated with J774A.1@HLPC, RBC@HLPC, HeLa@HLPC, and M@HLPC, in which the NPs were coated with distinct membrane materials from diverse cell lines (macrophage J774A.1, red blood cells (RBC), HeLa, and U251, respectively). As shown in Fig. 4b, U251 cells exhibited superior uptake of M@HLPC compared to other membrane-coated HLPCs, again confirming the parent cells-specific homing preference of M@HLPC. This parent cells-specific homing preference of M@HLPC could be attributed to the proteomics of present cell membrane, which owned several types of proteins with cell recognition function, such as CD44, cadherin-2, and zyxin^35^ (Supplementary Figs. 7, 8, Supplementary Table 1).

In addition to the improved uptake of M@HLPC by their parent cells, we next assessed the BBB penetration capacity of M@HLPC using a classic in vitro BBB model^36^. Briefly, human brain endothelial cells (hCMEC/D3) and a mixed layer of pericytes and astrocytes were cultured in the upper chamber of a Transwell™ apparatus, while human GBM tumor cells (U251) were cultured in the lower chamber (Fig. 4c). After the value of trans-endothelial electrical resistance (TEER) reached 150 Ω•cm^2^ (which indicated a successful BBB formation^37^), we added HLPC, J774A.1@HLPC, RBC@HLPC, HeLa@HLPC, or M@HLPC to the upper chamber. CLSM of hCMEC/D3 and the corresponding BBB layer imaging showed that the M@HLPC samples had the strongest fluorescence signals in the human brain endothelial cells in the upper chamber rather than in groups of uncoated HLPC or those coated with non-glioma cell membrane materials (Fig. 4d), which should be attributed to the transcytosis sourced from the specific recognition between coated U251 cell membrane and hCMEC/D3 cells (Supplementary Fig. 9). In the lower chamber, time-dependent quantification data also showed that M@HLPC exhibited the highest uptake efficiency by U251 cells (Fig. 4e). Taking 48 h as an example, the intracellular M@HLPC yielded at least 3.1-fold compared to that of other NPs. These in vitro penetration assays highlighted the strong potential for M@HLPC to cross the BBB and subsequently target glioma cells.

**Fig. 5:**
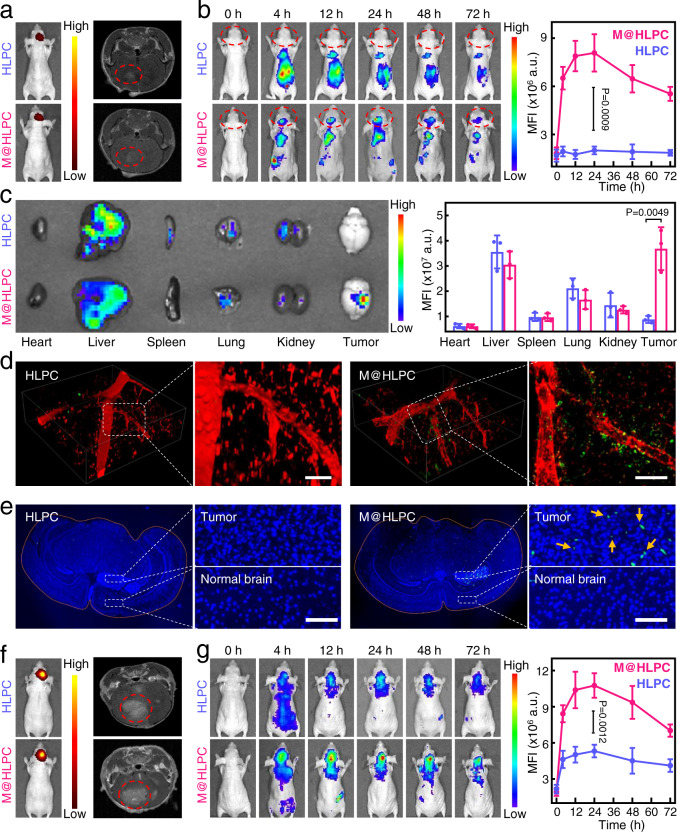
In vivo evaluation for the glioma-targeting of M@HLPC in orthotopic U251-luc glioma-bearing mice. **a** Representative bioluminescence images and MRI transverse section view of small-size glioma-bearing mice at 9 days’ post tumor cell inoculation. The mice were randomly divided into two groups and i.v. injected with HLPC or M@HLPC for the subsequent distribution analysis. Red circles: tumor regions. **b** In vivo distributions and the signal profiles of HLPC or M@HLPC in small-size U251-luc glioma-bearing Balb/c nude mice at different time points post i.v. injection. The fluorescence signals originate from Ce6 inside the formed NPs. **c** Representative ex vivo fluorescence images and corresponding quantitative fluorescence analysis of major organs and glioma tumors dissected from glioma-bearing mice at 24 h after i.v. injection with HLPC or M@HLPC. **d** In vivo two-photon images indicating the diffusion of HLPC and M@HLPC across the brain microvascular endothelial cells. Red: Tetramethylrhodamine isothiocyanate dextran-labeled blood vessels; green: NPs. Scale bar: 50 μm. **e** Representative fluorescence images of frozen brain sections at 24 h post NPs injection showed the effective accumulation of M@HLPC as yellow arrows indicated in the enlargement. Blue: Hoechst 33342-labeled nuclei; green: NPs. Scale bar: 50 μm. **f** Representative bioluminescence images and MRI transverse section view of large glioma-bearing mice at 18 days’ post U251 tumor cell inoculation. The mice were randomly divided into two groups and i.v. injected with HLPC or M@HLPC for the subsequent analysis of distributions. Red circles: tumor regions. **g** In vivo distributions and the signal profiles of HLPC or M@HLPC in large-size U251-luc glioma-bearing Balb/c nude mice at different time points post i.v. injection. The fluorescence signals originate from Ce6 inside the formed NPs. Quantitative data in (**b**, **c**, **g**) were presented as the mean ± SD, *n* = 1 experiment, *n* = 3 mice per group. Images were representative of three independent mice. *P* values were calculated by using two-tailed unpaired Student’s *t*-test. Source data are provided in the Source data file.

To determine whether such M@HLPC targeting performance also occurred in vivo, we inoculated U251-luc cells into the brain of Balb/c nude mice to form an orthotopic glioma model, and injected NPs into mice through the tail vein. Previous studies had reported that the intact structure and function of the BBB were mostly preserved at the early stage of glioma, and most nanomaterials could not traverse the BBB and accumulate in glioma for early diagnosis and therapy^37,38^. In order to explore M@HLPC accumulation performance in glioma at an early stage, we first used a small-sized xenograft glioma model (9 days after intracranial inoculation with U251-luc cells) for assessment. After the brain tumors were characterized using bioluminescence imaging and magnetic resonance imaging (MRI) (Fig. 5a), we then intravenously (i.v.) injected HLPC and M@HLPC. The results showed that the Ce6 signal was evident in the brains of the M@HLPC-treated but not the HLPC-treated mice (Fig. 5b). Further ex vivo imaging of the brains and other main organs also showed a unique signal in the brain tumor region of the M@HLPC-treated mice (Fig. 5c). Subsequently, we monitored BBB penetration by conducting two-photon intravital live imaging of the GBM model mice through a cranial window. We observed that high amount of M@HLPC had penetrated blood vessels and infiltrated into the glioma, whereas very few HLPC was detected inside the glioma (Fig. 5d). We also examined frozen brain sections: again, M@HLPC showed rich accumulation in the glioma area whereas almost no HLPC signal was detected in the glioma area (Fig. 5e). Similar results were also observed by constructing the M@HLPC with GL261-luc cell membrane and using corresponding orthotopic glioma model (Supplementary Fig. 10). These results demonstrated that the glioma cell membrane-coated NPs had the ability to traverse the slightly disrupted BBB and accumulate in glioma at an early stage.

To further investigate the targeting performances of M@HLPC in relatively larger glioma, a glioma model after 14 days of tumor cells intracranial inoculation was used. The bioluminescence imaging and MRI results demonstrated augmented tumor burden and enlarged tumor area in these U251-luc-based glioma models (Fig. 5f). At this advanced stage, the BBB was reported to undergo locally disruption by the rapid growth of glioma cells^39^. Under this circumstance, a few HLPC diffused through the disrupted BBB and entered into the brain tumors (Fig. 5g). Notably, much higher accumulation in the brain tumor was observed in the M@HLPC group at the same time intervals (Fig. 5g). Such a disparity was further confirmed by ex vivo imaging of the gliomas and major organs dissected from glioma-bearing mice (Supplementary Fig. 11). These findings clearly supported that coating HLPC with membrane materials from glioma cells elevated both their BBB-penetrating and glioma-targeting abilities.

### In vitro synergistic antitumor efficacy of M@HLPC

**Fig. 6:**
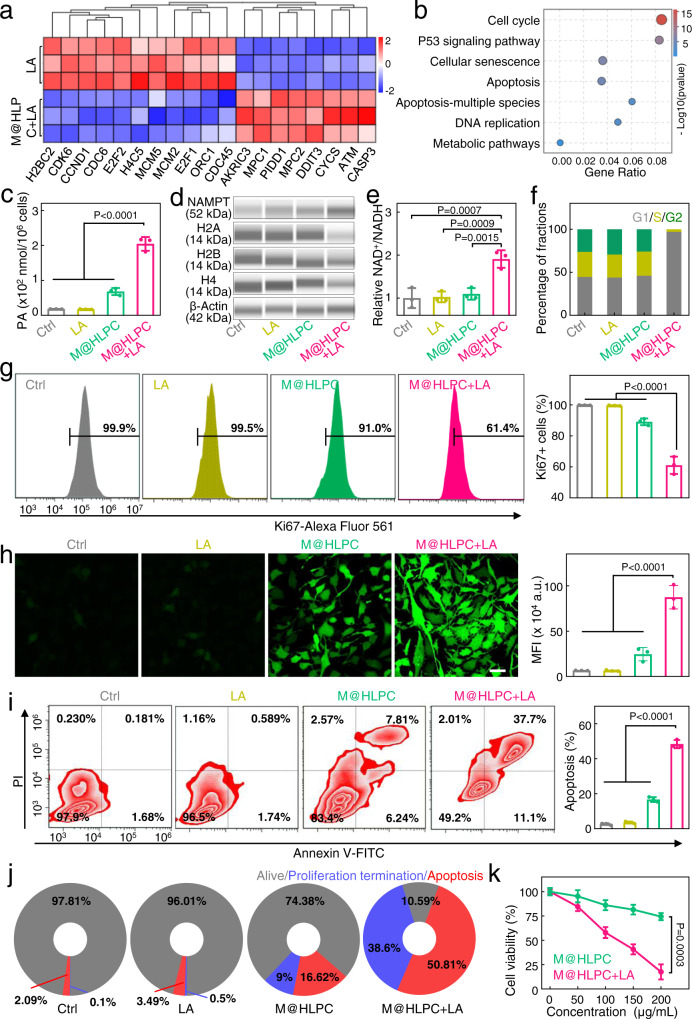
In vitro evaluation of the synergistic therapeutic effects of M@HLPC against hypoxic U251 cells. **a** Heat map showing the differentially expressed genes in M@HLPC + LA-treated and LA-treated U251 cells. Exogenous 5 mM LA was added to enhance the LA level in the cultured U251 cells. **b** Kyoto Encyclopedia of Genes and Genomes (KEGG) enrichment analysis of the differentially-expressed genes between M@HLPC + LA and LA treatment groups. **c** Quantitative analysis of PA concentration in U251 cells receiving the following different treatments: PBS, LA, M@HLPC, or M@HLPC + LA. **d** Intracellular NAMPT and histones (H2A, H2B, and H4) levels in U251 cells analyzed by ProteinSimple Wes^TM^ Capillary Western Blot analyzer. The original data were provided in Supplementary Fig. 14. **e** Analysis of the NAD^+^/NADH ratio in U251 cells using NAD^+^/NADH Assay Kit. **f** Cell-cycle progression with different treatments, determined by flow cytometry. G1, S, and G2 represented G1 phase, S phase, and G2 phase of cell division cycle, respectively. All cells were synchronized to the G1 phase with 1.5 mM HU before subjecting to the indicated treatments. **g** Flow cytometry and corresponding quantitative analysis of U251 cell proliferation by Ki67 staining. **h** Representative CLSM images and corresponding quantitative analysis of DCFH-DA for the generation of ^1^O_2_ in U251 cells receiving the indicated treatments. Scale bar: 50 μm. **i** Flow cytometry and corresponding quantitative analysis of the extent of U251 cell apoptosis by Annexin V/PI staining. **j** Proportions of U251 cells that were alive (gray), proliferation termination (blue), or apoptosis (red) after the indicated treatments. Data of proliferation termination and apoptosis were derived from (**g**), (**i**), respectively. **k** Relative cell viability of U251 cells after the indicated treatments for cultures supplemented with the indicated M@HLPC concentrations. LA: 5 mM. Quantitative data in (**c**), (**e**), (**g**–**i**), (**k**) were presented as the mean ± SD, *n* = 3 independent samples. The experiments in (**d**), (**h**) were repeated independently three times with similar results. *P* values were calculated by using one-way ANOVA. Source data are provided in the Source data file.

Next, we explored the antitumor activity of M@HLPC in vitro. To study the tumor cell inhibition mechanism of our M@HLPC, we first conducted RNA-sequencing (RNA-seq) analysis to identify differentially-expressed genes in U251 cells with M@HLPC treatment. In this experiment, to mimic the high level of LA in tumor microenvironment, 5 mM LA was exogenously added in the culture of both control and M@HLPC-treated cells. As shown in Fig. 6a, U251 cells with M@HLPC + LA treatment exhibited decreased expression of many genes with known proliferation-related functions (e.g., CDK6, E2F2, CDC6, and MCM2), but increased expression of apoptosis-related genes (CASP3, ATM, CYCS, and MPC1). Further Kyoto Encyclopedia of Genes and Genomes (KEGG) enrichment analysis indicated that the M@HLPC + LA treatment affected pathways related to cell proliferation or apoptosis (e.g., cell cycle, cellular senescence, apoptosis, and P53 signaling pathway) (Fig. 6b).

Focusing on the downregulated cell proliferation reflected by RNA-seq data, we considered that it was induced by the PA derived from LOX-catalyzed LA catabolism. In our experiment, elevated levels of PA were detected in the M@HLPC + LA-treated U251 cells (Fig. 6c), which encouraged us to explore their termination performance. Inspired by research suggesting that exogenous PA could terminate tumor cell proliferation through “NAMPT-NAD^+^-histone” axis (Supplementary Fig. 12), we systematically investigated the expression of NAMPT, NAD^+^, and histones (H2A, H2B, and H4) in U251 cells after different treatments. Here LA, M@HLPC, and exogenous PA groups (Supplementary Fig. 13) were also established for comparison with M@HLPC + LA. In order to truly reflect the effect of PA, a free radical scavenger singlet oxygen sensor green (SOSG) was added to all treated cells to absorb the produced ^1^O_2_ and neutralize its effect on cell proliferation. As expected, immunoblotting showed that the U251 cells treated with M@HLPC + LA and the exogenous PA (positive control) displayed a significant increase in NAMPT expression (Fig. 6d, Supplementary Figs. 13a, b, 14), which was attributed to the elevated MEF2C (Supplementary Fig. 15). While almost no change was observed in cells treated with LA and M@HLPC (with little PA being produced owing to the insufficient LA level in the cultured cells). Since NAMPT is required for NAD^+^ biosynthesis^40^, the up-regulated NAMPT increased the ratio of NAD^+^/NADH in both M@HLPC + LA and exogenous PA groups (Fig. 6e, Supplementary Fig. 13c). In addition, owing to the ability of increased NAMPT and NAD^+^ to histone gene expression^41^, significantly lower histone gene expression (H2A, H2B, and H4) was observed in the M@HLPC + LA and exogenous PA groups (Fig. 6d, Supplementary Figs. 13b, 16). Furthermore, considering the fact that histone gene expression is tightly coupled to cell cycle progression with synthesis and accumulation of histones restricted to the S phase^42^, we then assessed the impacts of M@HLPC + LA on the cell cycle. In this experiment, U251 cells were synchronized to the G1/S phase with hydroxyurea (HU), washed, and then released into media containing different formulations^43^. After different culturing time, cells were harvested for cell cycle analysis (Supplementary Fig. 17a), including G1 phase (G), S phase (S), and G2 phase (G2). As shown in Fig. 6f and Supplementary Fig. 17b, c, in the ctrl, LA, and M@HLPC groups, the cell cycle progressed normally to the S phase. However, in the presence of M@HLPC + LA and exogenous PA groups, cells were blocked in the G1/S phase and delayed entry into the S phase due to the repressive synthesis of histones. The delayed cell cycle ultimately resulted in the termination of cell proliferation (Fig. 6g, Supplementary Figs. 13d, 18).

With respect to another involved pathway—cell apoptosis, we supposed it was induced by the cytotoxic ^1^O_2_ produced via the chemiexcited PDT of M@HLPC. To explore this idea, we used 2′,7′-dichlorofluorescin diacetate (DCFH-DA) to measure the ^1^O_2_ level in U251 cells after different treatments. As shown in Fig. 6h, the same as to the PBS treatment, LA induced negligible changes in the DCFH-DA signal. The signal in U251 cells treated with M@HLPC was also weak due to the insufficient level of H_2_O_2_ in cultured cells. However, cells treated with M@HLPC + LA exhibited a much brighter signal, and this intensity was 3.56-times higher than that in the M@HLPC group, supporting the in situ LOX-catalyzed catabolism of LA into H_2_O_2_, as well as the subsequent production of ^1^O_2_ via chemiexcited PDT. Consistent with the known cytotoxic effects of ^1^O_2_, the greatest extent of apoptosis was also observed in the M@HLPC + LA group (Fig. 6i).

Owing to the synergistic effect of the metabolic therapy and chemiexcited PDT, we found that 38.6% and 50.81% of cells presented proliferation termination and apoptosis in the M@HLPC + LA group respectively, which were higher than that in the other groups (Fig. 6j). The abovementioned proliferation termination and apoptosis cooperatively resulted in the strongest decrease cell viability in M@HLPC + LA group, and we also found that the cell viability attenuation was concentration-dependent (Fig. 6k). Apart from M@HLPC, we also generated NPs lacking Ce6 (M@Hb-LOX-CPPO, “M@HLP”) and lacking LOX (M@Hb-CPPO-Ce6, “M@HPC”) for demonstrating their independent antitumor impact of the LA metabolic therapy and chemiexcited PDT (Supplementary Fig. 19). As a result, their toxicity to U251 cells was not as potent as that of M@HLPC (Supplementary Fig. 20).

### Therapeutic effects of M@HLPC on CDX model

**Fig. 7:**
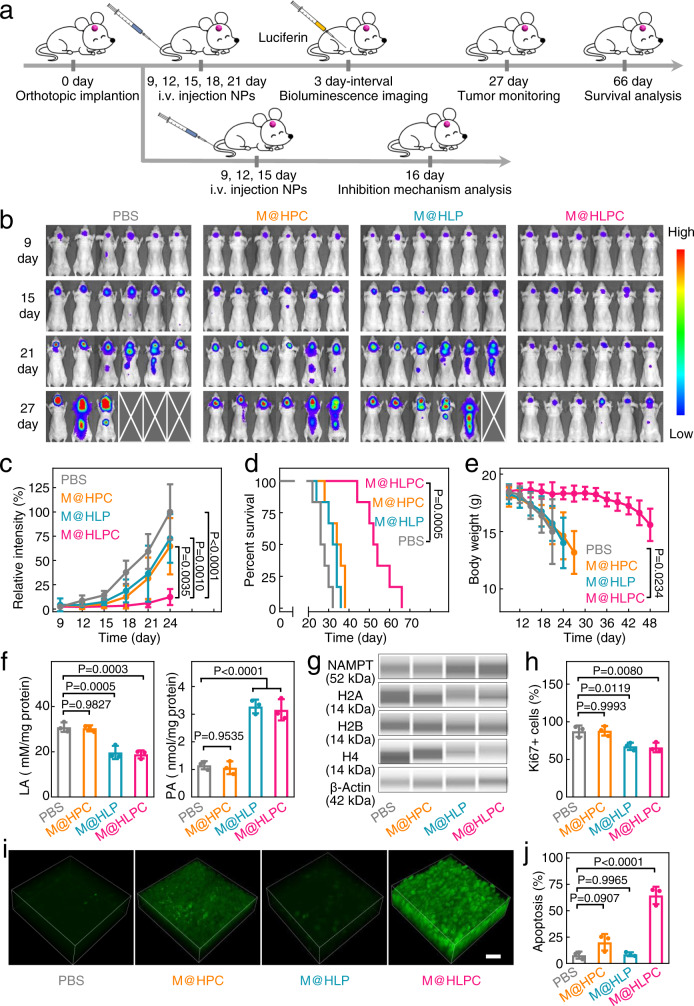
In vivo evaluation of the synergistic therapeutic effects of M@HLPC in U251-luc tumor-bearing mice. **a** Experimental design for evaluating the efficiency of tumor inhibition upon treatment with PBS, M@HLP, M@HPC, or M@HLPC in U251-luc tumor-bearing mice. **b** Bioluminescence images of U251-luc cells in glioma-bearing Balb/c nude mice receiving different treatments at the indicated time points. The blank area indicated that the corresponding mouse had died. **c** Quantification of the bioluminescence signal intensity from bioluminescence images on days 9, 12, 15, 18, 21, and 24. **d** Survival curves of the glioma-bearing mice receiving different treatments. **e** Body-weight change curves in mice receiving different treatments. **f** LA and PA concentrations in tumor tissue after i.v. injection of the indicated agents. **g** Intracellular NAMPT and histones (H2A, H2B, and H4) levels in tumor tissue. The original data were provided in Supplementary Fig. 24. **h** Quantification of cell proliferation (indicated by Ki67) in tumor tissues. **i** Representative two-photon fluorescence images of DCFH-DA for the generation of ^1^O_2_ in tumor tissue after the indicated treatments. Scale bar: 50 μm. **j** Quantification of cell apoptosis (indicated by TUNEL) in tumor tissues. Data in (**c**, **e**, **f**, **h**, **j**) were presented as the mean ± SD, *n* = 1 experiment, *n* = 6 mice per group in (**c**) and (**e**); *n* = 3 mice per group in (**f**), (**h**), (**j**). Images in **i** were representative of three independent mice. *P* values were calculated by using one-way ANOVA (**c**, **f**, **h**, **j**), two-tailed unpaired Student’s *t*-test (**e**) or Log-rank tests (**d**). Source data are provided in the Source data file.

Encouraged by the above results, we finally evaluated the in vivo therapeutic efficacy of various NPs. We constructed U251-luc xenograft mice and randomly divided them into various groups on day 9 post tumor cells injection (Fig. 7a). As uncoated HLPC could not traverse the slightly-disrupted BBB and accumulate in small-sized xenograft gliomas (Fig. 5a–e), these NPs exhibited negligible antitumor therapeutic effect (Supplementary Fig. 21). Focusing on membrane-coated NPs groups (M@HLP, metabolic therapy; M@HPC, chemiexcited PDT; and M@HLPC, metabolic therapy synergized with chemiexcited PDT), bioluminescence imaging throughout the treatment course revealed that M@HLPC conferred the strongest antitumor effect (Fig. 7b). Quantitative analysis on day 24 showed that the tumor growth inhibition (TGI) of M@HLPC was 87.8%, which was more effective than M@HLP (27.7%) and M@HPC treatments (35.2%) (Fig. 7c). Consistently, survival curves showed that treatment with M@HLPC markedly increased survival rates to a median 53 days, significantly longer than that achieved with PBS (28 days), M@HPC (36 days), and M@HLP (34 days) (Fig. 7d). We also noted that the excellent TGI conferred by M@HLPC was accompanied by a delayed body-weight loss. The body weight in M@HLPC-treated mice almost did no change (about 18.8 g) within 33 days, while the body-weight in other groups was rapidly decreased to 14.0 g within a few days (Fig. 7e). In addition, no abnormal signals for blood physiological or biochemical indexes were observed in the M@HLPC-treated mice (Supplementary Fig. 22). Hematoxylin and eosin (H&E) staining of organ sections from the heart, liver, spleen, lung, or kidney showed no lesions or other abnormalities (Supplementary Fig. 23), supporting the safety of the M@HLPC treatment.

Above outperformed antitumor efficacy highlighted the synergetic effect of metabolic therapy and chemiexcited PDT. To gain a deeper insight into the inhibition mechanism, we investigated each of the two abovementioned effects separately. For the former, the LA and PA concentrations as well as the expression levels of NAMPT and histones in the tumor tissues were evaluated. In this experiment, 5 μL SOSG was intratumorally injected to consume the produced ^1^O_2_ and exclude the effect of chemiexcited PDT. As shown in Fig. 7f, compared with the PBS and M@HPC-treated animals, the M@HLP and M@HLPC groups (LOX containing groups) displayed a decreased LA concentration and an increased PA concentration. These altered metabolite levels led to the expected activation of NAMPT and subsequent repression of histones (H2A, H2B, and H4) (Fig. 7g, Supplementary Fig. 24). The different histone gene expression inhibition resulted in distinguishing termination of tumor cell proliferation, which was detected by the expression level of Ki67 (Fig. 7h, Supplementary Fig. 25). For the aspect of chemiexcited PDT, we estimated ^1^O_2_ production in tumors using in situ two-photon fluorescence microscopy imaging after intratumoral administration of DCFH-DA. As shown in Fig. 7i, the signal for ^1^O_2_ was barely observed in the PBS group and Ce6 absent M@HLP group. As for the M@HPC group, a weak signal was detected due to the inherent H_2_O_2_ inside the tumor. In contrast, the M@HLPC group showed strong ^1^O_2_ signal and thus induced the highest degree of apoptosis (Fig. 7j, Supplementary Fig. 25).

### Construction of hM@HLPC and verification of therapeutic efficacy in PDX model

**Fig. 8:**
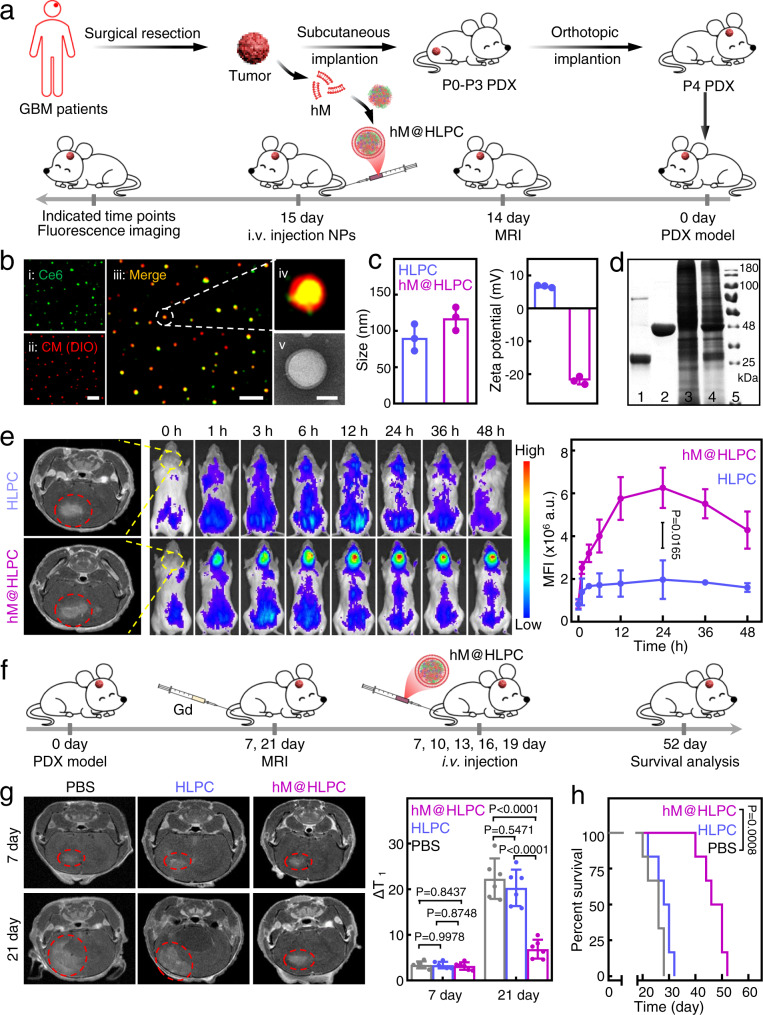
Evaluation of the synergistic therapeutic effect of hM@HLPC in patient-derived xenograft (PDX) model. **a** Schematic illustration of the experimental design for humanized hM@HLPC construction, PDX model construction, and evaluation of the BBB penetration and tumor-targeting ability of hM@HLPC. **b** Super-resolution fluorescence and TEM images of hM@HLPC. i–iv: green, Ce6 (inside the formed HLPC); red, DIO-labeled the coated hM. Scale bar: 2 μm. v: hM@HLPC were negatively stained with 2% phosphotungstic acid solution for TEM imaging. Scale bar: 50 nm. Super-resolution fluorescence and TEM images indicated the presence of hM coating on HLPC. **c** Size and zeta potential of HLPC and hM@HLPC analyzed by dynamic light scattering (DLS). DLS showed an increase in the hydrodynamic diameter and reversed surface charge between the HLPC and hM@HLPC samples, indicating successful coating of the HLPC with hM. **d** SDS-PAGE analysis of (1) Hb, (2) LOX, (3) hM, (4) hM@HLPC, and (5) Marker. The copresence of Hb, LOX, and hM in hM@HLPC demonstrated the successful hM coating. **e** MRI transverse section view of GBM PDX tumor-bearing mice before treatment (left), in vivo distributions (middle), and the signal profiles (right) of HLPC or hM@HLPC in GBM PDX tumor-bearing mice at different time points after i.v. injection. **f** Schematic illustration for the evaluation of the tumor inhibition effects of hM@HLPC against the PDX model. **g** MRI transverse section view of GBM PDX tumor-bearing mice and corresponding quantification of the T1-weighted MRI signal of the tumor site at 7 and 21 days after i.v. injection of PBS, HLPC, or hM@HLPC. **h** Survival curves of GBM PDX tumor-bearing mice in different groups. Quantitative data in (**c**, **e**, **g**) were presented as the mean ± SD, *n* = 3 independent samples in (**c**); *n* = 1 experiment, *n* = 3 mice per group in (**e**); *n* = 1 experiment, *n* = 6 mice per group in (**g**). The experiments in (**b**, **d**) were repeated independently three times with similar results. *P* values were calculated by using two-tailed unpaired Student’s *t*-tests (**e**), one-way ANOVA (**g**), or Log-rank tests (**h**). Source data are provided in the Source data file.

To further investigate the clinical applicability of our biomimetic nanodrug, we established a humanized nanodrug platform (hM@HLPC) and evaluated its therapeutic effect in a PDX model (Fig. 8a). Briefly, the primary tumor sample was resected from a GBM patient. One part of the tumor was dissected into tiny pieces and triturated into single-cell suspension, after which we prepared the cell membranes of the human tumor tissue (hM) using the same protocol as the aforementioned U251 cells. The prepared hM was subsequently coextruded with HLPC for the hM@HLPC construction. The colocalization of HLPC and hM signals in the super-resolution fluorescence images (Fig. 8b, i–iv), the membrane layer around HLPC in the TEM image (Fig. 8b, v), the increased particle size and reversed zeta potential (Fig. 8c) together demonstrated the successful hM coating. Moreover, the presence of multiple components of Hb, LOX, and hM in the SDS-PAGE image of hM@HLPC demonstrated the successful construction of versatile NPs (Fig. 8d).

After verifying the BBB-penetrating capacity (Supplementary Fig. 26) and synergistic antitumor mechanism of hM@HLPC in vitro (Supplementary Fig. 27), we continued to investigate the in vivo performance. To this end, another part of patient tumor sample was subcutaneously transplanted into the axilla of NOD.Cg-Prkdc^scid^ Il2rg^tm1Vst^/Vst (NPG) mice. After engraftment for three passages, the tumor tissue was dissociated into single cells using trypsin-EDTA. Then the cells were stereotactically injected into the brain of NPG mice to establish the GBM PDX model. After confirming the successful formation of the brain tumor at day 14 (Fig. 8e, left panel), HLPC or hM@HLPC were i.v. injected into tumor-bearing mice at day 15 for evaluating the brain tumor-targeting ability. As shown in Fig. 8e (middle and right panel), a brighter fluorescence signal was observed in the brains of hM@HLPC group, and the intensity was 3.1-times higher than that of the HLPC at 24 h post-injection. Similar differences in signal strength were also observed in excised tissues (Supplementary Fig. 28), clearly supporting the superiority of the resected tumor-derived membrane of hM@HLPC in BBB-penetrating capacity and homotypic tumor targeting. For the final evaluation of tumor therapeutic efficiency, hM@HLPC were i.v. injected at days 7, 10, 13, 16, and 19 after PDX model construction, and MRI was used to detect brain tumor burden (Fig. 8f). Compared with the PBS and HLPC groups, the tumors of the hM@HLPC mice were significantly smaller at day 21 (Fig. 8g) and the median survival time of the hM@HLPC-treated mice was significantly prolonged to 48 days, while the values in PBS and HLPC groups were only 26 and 29 days, respectively (Fig. 8h). These data strongly suggested that hM sourced from resected GBM tissues could be used to construct personalized nanodrug (hM@HLPC), endowing them with enhanced glioma-targeting efficacy and significant antitumor performance in a matched PDX model.

## Discussion

In summary, we have successfully developed M@HLPC, a biomimetic targeted drug delivery and synergistically therapeutic system for the effective treatment of GBM. Exploiting the strong interaction between CPPO and proteins, we used a one-pot self-assembly strategy for the construction of HLPC, which were then encapsulated with membranes prepared from glioma cells to generate the biomimetic M@HLPC. The result M@HLPC displayed excellent stability and efficient BBB penetration and tumor targeting. Upon reaching tumor sites, the combined activities of the metabolic therapy and chemiexcited PDT agents conferred strong tumor inhibition in murine xenograft tumor model. We extended our study with a successful example of a personalized therapy using hM@HLPC against patient-derived tumor model, thereby highlighting the strong translational potential of our hM@HLPC system to develop clinically relevant GBM therapies.

Currently, surgical tumor resection is the primary clinical treatment strategy for nearly 75% of GBM patients^44^. However, due to the highly infiltrative and invasive nature of glioma cells, the infiltrating tumor cells inside the normal brain parenchyma cannot be completely removed by surgical resection. These residual tumor cells easily lead to the recurrence of gliomas. Therefore, targeting and eliminating the infiltrating glioma cells in GBM treatment is of utmost importance^45–47^. Considering the clinical resected tumor tissue that carries patient personalized molecules is usually underutilized (except for the biopsy) and membrane materials can be conveniently obtained from resected glioma tissue, we proposed utilizing the membrane materials from GBM tissue to construct personalized nanomedicine. First, multiple surface adhesion molecules (such as E-cadherin and epithelial cell adhesion molecules) on the tissue membrane may endow these personalized nanomedicines with the homotypic tumor self-recognition ability. In this context, the infiltrating gliomas cells missed by surgery can be targeted and cleared by the personalized membrane-based nanomedicine, thus preventing post-surgical tumor recurrence. Furthermore, the resected GBM tissue can be utilized for ex vivo proliferation with culture medium containing specific factors (i.e., Wnt3A, hFGF10, hEGF)^48,49^, which can provide for preparing personalized nanomedicine. In addition, the membrane materials can be reserved in −80 °C for long time and be re-thawed for the preparation of biomimetic nanomedicine once the tumor recurrence sign emerges. Above benefits illuminate the clinical translation potential of our tumor tissue cell membrane-based personalized system for the treatment of recurrent tumors.

Regarding future development of these technologies, it should be noted that many types of tumors display elevated LA content (including lung, gastric, and pancreatic cancer)^12^, thus, the synergistic combination of LA metabolic therapy and chemiexcited PDT that we showed in the present study may be suitable to treat a wide range of cancers. Given that the intratumoral level of LA has been reported higher than that of glucose^26,50^, the direct utilization of LA rather than glucose may also exhibit the advantage of owning more adequate fuel. It is worth mentioning that LA has been reported to induce the polarization of M2 macrophages^51^. We did not observe the obvious polarization of M2 macrophages in our experiment. This is acceptable due to the chasm existing in the TME between GBM and other solid tumors. The decrement of LA in the TME may insufficiently induce the polarization of the large population of macrophages that typically infiltrate GBM tumors or perhaps there was some impact by the elevated PA content resulting from LOX-catalyzed LA catabolism^52,53^.

More specifically, the therapeutic effects of the traditional photodynamic therapy (PDT) for GBM are limited by the depth of penetration in the brain upon laser irradiation^54,55^. In our system, the loaded Ce6 could be activated by the chemical energy produced from the reaction between CPPO and H_2_O_2_, achieving chemiexcited PDT without light excitation. Looking beyond the CPPO and Ce6 cargo that we loaded for chemiexcited PDT, it should be possible to flexibly load our nanocarrier system with other hydrophobic photosensitizers (such as protoporphyrin IX) to generate ^1^O_2_ through PDT against superficial tumors. With regard to the co-delivered LOX, many other enzymes, small molecular inhibitors, and siRNAs provide various replaceable candidates to extend the applications of metabolic therapy. For example, LOX can be replaced by glucose oxidase, which can catalyze the oxidation of glucose and produce H_2_O_2_ for the potential synergy between metabolic therapy and the chemiexcited PDT. Moreover, the proposed self-aggregating and encapsulation features of the proteins-based NPs exhibited an easily-manipulative and efficient paradigm for constructing a versatile nanocarrier platform. In this aspect, different types of proteins, small nanoparticles, and even their combinations, can also be easily incorporated. In addition, loading additional hydrophilic or hydrophobic drugs (e.g., drugs like paclitaxel, doxorubicin, and indocyanine green) is a simple task for our encapsulation system. The flexibility and efficiency mentioned above not only guarantees the convenience and safety, but also enables us to achieve desired antitumor efficiency for diverse therapeutic applications.

## Methods

### Study approval

All animal experiments were performed in accordance with the Guide for the Care and Use of Laboratory Animals (China, GB/T 35892-2018). The animal protocol was approved by the Institutional Animal Care and Use Committees at the Institute of Process Engineering, Chinese Academy of Sciences (approval ID: IPEAECA2019701). A primary tumor sample was acquired from GBM volunteer (age 46, female), who was recruited through the protocol at the Shenzhen Second People’s Hospital. The human protocol was approved by the Ethics Committee of Shenzhen Second People’s Hospital (approval ID: 20210507004-FS01) and informed consent was obtained from the participant.

### Materials and reagents

Hemoglobin (Hb), 9,10-Anthracenediyl-bis(methylene) dimalonic acid (ABDA), and bis(2,4,5-trichloro-6-carbopentoxyphenyl) oxalate (CPPO) were obtained from Sigma-Aldrich (St. Louis, MO, USA). Lactate oxidase (LOX) was purchased from Shanghai Yuanye Bio-Technology (Shanghai, China). Chlorin e6 (Ce6) was purchased from J&K Scientific Ltd (Beijing, China). RPMI 1640 medium, Dulbecco’s modified Eagle’s medium (DMEM), and fetal bovine serum (FBS) were obtained from Gibco Life Technologies (AG, Switzerland). Rhodamine phalloidin was obtained from Invitrogen (USA). Trypsin-EDTA and penicillin-streptomycin, were purchased from Corning life sciences (Wujiang, China). Hoechst 33342, calcein-AM/propidium iodide (PI), Apoptosis Analysis Kit, and 2′,7′-dichlorofluorescin diacetate (DCFH-DA) were purchased from Solarbio^®^ life sciences (Beijing, China). Cell cycle Analysis Kit was obtained from Beyotime (Beijing, China). The antibodies and corresponding dilution ratio were listed in Supplementary Table 2. The siRNA sequences (Sangon Biotech, Shanghai) were listed in Supplementary Table 3. pcDNA3.1-MEF2C (KWC20220217AZGYQ-PC01) and pcDNA3.1-NAMPT (KWC20220217AZGYQ-PC02) were constructed by Hanbio Biotech (Shanghai, China).

### Cell culture

Human cervical carcinoma cell (HeLa, catalog no. CRM-CCL-2^TM^) and mouse mononuclear macrophages (J774A.1, catalog no. TIB-67^TM^) were obtained from the American Type Culture Collection (ATCC). The human brain endothelial cell (hCMEC/D3, catalog no. 337728) was purchased from BeNa Culture Collection. The luciferase-transfected glioblastoma cell lines (U251-luc, catalog no. CBP30207L and GL261-luc, catalog no. iCell-0059a) were maintained in our laboratory (Shenzhen Second People’s Hospital, Shenzhen, China). Human astrocytes (catalog no. 1800) and brain vascular pericytes (catalog no. 1200) were purchased from Sciencell. The cells have been confirmed without mycoplasma contamination by mycoplasma detection kit (Beijing Solarbio Science & Technology Co., Ltd., CA1080). All types of cells were cultured in DMEM and supplemented with 10% FBS and 1% (v/v) penicillin-streptomycin. The normoxic cells were incubated in a humidified atmosphere with 5% CO_2_ and the hypoxia cells were maintained in an incubator with 1% O_2_, 5% CO_2_, and 94% N_2_. Red blood cells (RBC) were obtained from the peripheral blood of mice.

### Animals and tumor models

#### Animals

Four-week-old female Balb/c nude (catalog no. 13001A) and C57BL/6 (catalog no. 11001A) mice were purchased from Vital River Laboratories (Beijing, China). Four-week-old female NOD.Cg-Prkdc^scid^ Il2rg^tm1Vst^/Vst (NPG, catalog no. NPG-1) mice were purchased from Shanghai Model Organisms Center, Inc. All the model mice were raised in a standard environmentally controlled room (23 °C, with 55 ± 5% humidity and under a 12 h/12 h light/dark cycle).

#### Glioma orthotopic implantation tumor model

Balb/c nude and C57BL/6 mice were immobilized on a stereotactic frame (RWD Life Science, Shenzhen) after anesthetized with 1% pentobarbital sodium. Then, 3 µL of U251-luc and GL261-luc cells (3 × 10^5^) were stereotactically injected into the brain parenchyma (bright lateral: 2.0 mm, bregma: 1.8 mm, depth: 3 mm) of Balb/c nude or C57BL/6 mice, respectively. The growth of intracranial glioma cells was monitored by bioluminescence imaging (IVIS imaging system, PerkinElmer, USA, version 4.5.5).

#### Patient-derived xenograft (PDX) tumor model

A primary tumor sample was resected from a GBM patient. After transport from pathology to laboratory in HBSS at room temperature, the tumor was divided in two parts. One part of the tumor was subcutaneous transplanted into the axilla of the NPG mice and engrafted for three passages (another part for the construction of hM@HLPC). After being resected, the tumor tissue was cut into tiny pieces and dissociated into single-cells by using trypsin-EDTA. Then the cells were stereotactically injected into the brain parenchyma of the NPG mice using the same protocol as the aforementioned “glioma orthotopic implantation tumor model”. The growth of tumors was monitored by magnetic resonance imaging (MRI, BioSpec 70/20 USR, Bruker, Germany, version 6.01).

### Sample collection and immunohistochemistry

#### Patient samples

Twenty-two cases of diffuse astrocytoma and oligodendroglioma (grade II), 21 cases of anaplastic astrocytoma and anaplastic oligodendroglioma (grade III), and 23 cases of glioblastoma multiforme (grade IV) were selected from Shenzhen Second People’s Hospital. Resected glioma samples were obtained from patients with informed consent and were reviewed by the pathologist and surgeon. Pathologist classified the type and grade of the gliomas in accordance with the WHO histological grading of central nervous system tumors.

#### Mouse samples

The brains of healthy and tumor-bearing mice (14 days after intracranial inoculation with U251-luc cells) were collected and sectioned into ten-μm-thick slices.

#### Immunohistochemistry (IHC)

Ten-μm-thick tissue sections (human and mice) were de-waxed and rehydrated through graded alcohols. Antigen retrieval was carried out using Dako PT link (Dako/Agilent Technologies, Santa Clara, CA). IHC staining of individual markers lactate dehydrogenase A (LDHA), monocarboxylate transporter 4 (MCT4), or proliferation marker (Ki67) was performed using EnVision™ G|2 Doublestain System, rabbit/mouse (DAB/Permanent Red) kit (Dako/Agilent Technologies, Santa Clara, CA), according to the manufacturer’s instructions. Slices were imaged by an automatic multispectral imaging system (Vectra II, PerkinElmer version 2.0.7.1).

#### Database

cBioportal (URL: http://www.cbioportal.org/) was used to assess the dataset of low-grade gliomas (LGG) and high-grade gliomas (HGG) from The Cancer Genome Atlas (TCGA). Freely accessible server, oncoLnc tool (URL: www.oncol.nc.org) generated the Kaplan–Meier plots for the studied genes using the low and high-expressing LDHA and MCT4 that are publicly available in TCGA database.

### Preparation of HLPC

3 mL of Hb (0.7 mg/mL) and 70 μL LOX (5 mg/mL) were placed in a 50 mL sterile tube. Then 16.8 μL CPPO (19.4 mM) and 5.04 μL Ce6 (38.8 mM) in DMSO were slowly added during sonication. After 15 min, the resulting solution was centrifuged for 10 min (4 °C, 15,000 × *g*), and the precipitation was purified with centrifugal filters (Centrifugal filter devices, 100 kD, Millipore) to remove free drugs. The concentration of protein (Hb and LOX) was measured by BCA assay. To determine the Hb content in HLPC, Fe concentration in free Hb or HLPC was measured by inductively coupled plasma optical emission spectrometer (ICP-OES, iCAP 6300, version 2.8.0.89). The loading efficiency of CPPO and Ce6 was determined by UV/vis absorption spectra.

### Preparation of M@HLPC

The cell membrane materials (M) were prepared according to our reported procedure^34^. Briefly, U251 cells with 1% EDTA-free protease inhibitor were disrupted using an IKAT18 basic homogenizer (IKA, Germany) for 1 h. Then, the supernatants were collected by ultracentrifuging (10,000 × *g*, 10 min) and laid on a discontinuous sucrose density gradient. After ultracentrifugation (28,000 rpm, 2 h), the 30% band was collected and ultracentrifuged at 28,000 rpm for 30 min to obtain M. For the preparation of M@HLPC, the collected M was first extruded through a 200 nm porous membrane, and then HLPC was added (1:3 in mass). After coextruding through a 100 nm porous membrane for 10 times, the production was centrifuged at 4 °C for 10 min (10,000 × *g*) to remove excessive M and got the final M coated HLPC (denoted M@HLPC). The M@LPC (lacking Hb-O_2_), M@HPC (lacking LOX), and M@HLP (lacking Ce6) were prepared similar with that of M@HLPC.

In order to obtain de-proteinated cell membrane-coated M(-)@HLPC, the cell membrane materials (M) were successively treated with 0.5 U/mg proteinase K at 37 °C for 8 h and trypsin EDTA 0.01% overnight. After proteolysis, the coating of M(-)@HLPC was performed as that of M@HLPC.

### Characterization of HLPC and M@HLPC

The interaction between each sub-component in HLPC was measured by microscale thermophoresis (MST, NT.115, Nanotemper, Germany, version 2.3). The size and zeta potential of HLPC and M@HLPC were measured by using a nanoparticle tracking analysis (NTA, Particle Metrix, Germany, version 8.05.04) at 25 °C. Transmission electron microscope (TEM) of HLPC and M@HLPC were recorded on JEOL JEM-1400 (Japan, version 1.7.18.2349). The samples dispersed in PBS were dropped onto 200 mesh C-coated Cu grid (Beijing Zhongjingkeyi Technology) prior to recording the TEM images. UV/vis absorption and fluorescence spectra were obtained with an automatic microplate reader (Tecan Infinite M200, version 1.6.19.2). To verify the M on the HLPC particles by fluorescence imaging, the HLPC and M@HLPC were fixed on glass-bottom dishes at 37 °C for 1 h and washed with PBS, followed by incubation with 3,3′-dioctadecyloxacarbocyanine perchlorate (DIO) for 1 h. Then, the nanoparticles (NPs) were viewed by confocal laser scanning microscopy (CLSM, NIKON, version 5.20.00). To monitor the colloid stability, M@HLPC were dispersed in PBS or FBS (10%) and kept at 4 °C. The size and zeta potential were measured via a ZetaSizer (NANO ZS, Malvern, version 7.12) every other day. To study the spectroscopic stability, the free Ce6 and M@HLPC were dispersed in PBS and kept at 4 °C or 37 °C, the UV/vis absorption spectra of Ce6 were measured every other day.

#### Measurement of oxygen release profile

The oxygen (O_2_) release profiles of free Hb, M@LPC (lacking Hb-O_2_), and M@HLPC in hypoxic PBS were measured using a dissolved oxygen meter. Briefly, 8 mg of free Hb and M@HLPC containing the identical amount of Hb were dispersed in 200 μL of PBS and purged with O_2_ for 15 min. After that, the oxygen-saturated samples were slowly injected into 1.8 mL deoxy PBS solution (purged with nitrogen for 10 min and sealed with liquid paraffin). The concentration of dissolved O_2_ was recorded with time.

#### Lactate oxidation activity of M@HLPC

6.7 µg/mL free LOX (dissolved in PBS with normal O_2_), 100 µg/mL M@LPC (lacking Hb-O_2_), M@HPC (lacking LOX), and M@HLPC (equal concentration of LOX: 6.7 µg/mL) dissolved in hypoxic PBS was mixed with LA (5.0 mM) at 37 °C. The catalytic processes of lactate oxidation were analyzed by monitoring the concentration changes of the substrate lactate (LA) and the products of pyruvic acid (PA) according to the supplier’s operating instructions of Lactate Assay Kit (Abcam) and Pyruvate Assay Kit (Sigma-Aldrich).

#### Detection of singlet oxygen (^1^O_2_)

To verify the involvement of ^1^O_2_ in the fluorescence increment of ABDA, 1 mg/mL M@LPC (lacking Hb-O_2_), M@HPC (lacking LOX), M@HLP (lacking Ce6), and M@HLPC was suspended in hypoxic PBS containing 10 μM ABDA dye, followed by addition of different concentrations of LA. After incubated at 37 °C for 12 h, the fluorescence of ABDA (upon excitation at 378 nm) was measured using the automatic microplate reader. ^1^O_2_ generation was also verified via electron spin resonance by employing the radical scavenger TEMPO. Briefly, 10 μL TEMPO was added into 100 µL of the indicated samples at 37 °C. After incubated for 12 h, the samples were analyzed via ESR.

### In vitro homologous targeting capability of M@HLPC

#### Specific uptake performances of M@HLPC

To investigate the uptake of HLPC and M@HLPC by U251 and HeLa cells, the cells were seeded into glass-bottom dishes and incubated for 24 h, and then treated with HLPC and M@HLPC (100 μg/mL) for 1 h. For microscopy imaging, the nuclei and cell membrane were stained with Hoechst 33342 and Rhodamine phalloidin, respectively, and imaged by CLSM. Hoechst 33342 was excited with UV, emitting 450–500 nm fluorescence. Rhodamine phalloidin was excited with a 561 nm laser, emitting 580–630 nm fluorescence. The NPs (Ce6 signal) were excited using a 488 nm laser, and emission was collected in the range of 630–700 nm. For flow cytometry analysis, the cells were seeded into 24-well plates and treated as above. The collected cells were analyzed by flow cytometry (Beckman coulter, CytoFLEX, version 2.3.1.22).

To study the uptake of different membrane-modified HLPC by U251 cells, U251 cells were seeded into glass-bottom dishes or 24-well plates for 24 h. After separately incubated with J774A.1@HLPC, RBC@HLPC, HeLa@HLPC, and M@HLPC (in which NPs were coated with distinct membrane materials from diverse cell lines, including J774A.1, RBC, HeLa, and U251) for 1 h, the cells were analyzed by CLSM or collected for flow cytometry.

#### Proteomic analysis

Cell membranes harvested from U251 and primary healthy astrocytes were prepared for LC-MS/MS analysis. In detail, cell membranes were frozen in liquid N_2_ and the protein was isolated by SDT (4% SDS, 100 mM Tris/HCl pH 7.6, 0.1 M DTT) lysis method. After quantifying by BCA, the peptide samples were made by enzymolysis with filter-aided proteome preparation and desalination with C18 Cartridge. Then, the extracted peptides were lyophilized and re-dissolved in 10 μL of 0.1% formic acid. For LC-MS/MS analysis, the peptide mixtures were retained on a C18-reversed phase column (15 cm length, 150 μm i.d.) packed with ReproSil-Pur C18-AQ resin (1.9 μm, 100 Å, Dr. Maisch GmbH, Germany) using the nanoLC Utimate 3000 system. The sample was separated within a 60 min linear gradient at a flow rate of 600 nL/min. Mobile phase A (99.5% water and 0.5% formic acid) and mobile phase B (80% acetonitrile and 20% 0.1% formic acid in water) were used. The elution gradient was as follows: from 4% to 10% B for 5 min, from 10% to 22% B for 80 min, from 22% to 40% B for 25 min, from 40% to 95% B for 5 min, and from 95% to 95% B for 5 min. The nanoLC was directly interfaced with the Q Exactive™ Hybrid Quadrupole-Orbitrap™ Mass Spectrometer (Thermo Fisher Scientific). An electrospray voltage of 2.0 kV was employed. In MS1, the resolution was 70,000, and the maximum injection time was 20 ms. In MS2, the resolution was 17,500, and the maximum injection time was 60 ms. The raw MS files were analyzed and searched against the target protein database based on the species of the samples using Proteome Discoverer 2.5. Both LC-MS/MS analysis and data analysis were done by Beijing Biotech-Pack Scientific Co. Ltd (China).

#### Blocking assay

M@HLPC was pre-incubated with 10 µg/mL antibodies (anti-CD44, anti-cadherin-2, or anti-zyxin) for 1 h. After wash, the pretreated M@HLPC were added to the U251 cells and incubated another 1 h. Then, the cells were collected and analyzed by flow cytometry.

#### In vitro BBB penetration

Human brain endothelial cell hCMEC/D3 cells, human brain pericytes, and astrocytes were used to generate an in vitro blood–brain barrier (BBB) model as previously reported^36^. Monolayer of hCMEC/D3 cells and a mixed layer of pericytes and astrocytes (1 × 10^5^ cells/well) were separately plated on the top and reverse side of 0.4-μm pore size transwell membrane (24 mm Transwell™, Corning), which were pre-coated with gelatin first. U251 (1 × 10^3^ cells/well) were cultured in the lower chamber. After the trans-endothelial electrical resistance (TEER) of this model reached 150 Ω·cm^2^, HLPC, J774A.1@HLPC, RBC@HLPC, HeLa@HLPC, M(-)HLPC, and M@HLPC (100 μg) in fresh culture media were added to the top chamber. hCMEC/D3 cells (the cell membrane was stained with DIO), U251 cells (the nuclei were stained with Hoechst 33342) as well as XZ images of the transwell models, were individually captured by CLSM (NIKON, version 5.20.00) at 24 h incubation. The internalization of the above-indicated NPs by U251 cells was also analyzed using flow cytometry based on Ce6 fluorescence.

#### BBB crossing mechanism

The expression of VCAM1 on the hCMEC/D3 cells was analyzed by flow cytometry. For blocking experiments, hCMEC/D3 cells in the upper chamber were treated with different of inhibitors before adding M@HLPC. The uptake efficiency was determined by collecting samples for flow cytometry analysis.

### In vivo fluorescence imaging

U251 or GL261-luc orthotopic tumor-bearing mice were randomly divided into two groups (3 mice per group) and injected (i.v.) with HLPC or M@HLPC (200 μL, Ce6: 1 mg/kg). Fluorescence imaging was taken at different time intervals using an IVIS imaging system with a 670 nm excitation wavelength and a 720 nm filter to collect the Ce6 signals. In the ex vivo imaging experiments, the mice were sacrificed at 24 h post-injection. The major organs (heart, liver, spleen, lung, kidney, and brain) were collected and imaged immediately. For histological evaluation, dissected brains were frozen in OCT tissue compound on dry ice and sectioned into 10-μm slices. After stained with Hoechst 33342, the fluorescence images of all sections were acquired on automatic multispectral imaging system.

The in vivo targeting behavior was also conducted using a multiphoton laser confocal microscope (Leica TCS SP8, Germany, version 3.0) at 24 h post-injection of HLPC and M@HLPC. To fashion a cranial window, the skull was thinned away using a sterile stainless steel 2 mm diameter cylindrical drill bit attached to a high-speed hand drill until the underlying dura mater was exposed.

### In vitro synergistic antitumor efficacy of M@HLPC

#### RNA sequencing (RNA-seq) by Illumina HiSeq

After hypoxic U251 cells were treated with LA (5 mM) or M@HLPC + LA (5 mM) for 24 h, total RNA was extracted from cells using TRIzol (Invitrogen) and the quality was examined using Agilent 2100 Bioanalyzer (Agilent Technologies) according to the manufacturer’s instructions. Library construction, sequencing and bioinformatics analysis were done by Genedenovo Biotechnology Co., Ltd (Guangzhou, China). In detail, the obtained total mRNA was enriched by Oligo (dT) beads and fragmented into short fragments using the fragmentation buffer. Then, the short fragments were reversely transcribed into cDNA with primers. After being purified using a QiaQuick PCR Extraction kit, the cDNA products were sequenced using the Illumina HiSeq 2500 platform. The differentially expressed RNAs were identified by the fold-change method after castration using the R package DEGseq.

To verify the LA metabolic therapy effect, hypoxic U251 cells were cultured in 96-well plates and treated with PBS, LA (5 mM), M@HLPC, M@HLPC + LA (5 mM) or sodium pyruvate (5 mM, positive control). 10 μM free radical scavenger singlet oxygen sensor green (SOSG) was also added to consume the produced ^1^O_2_. After 24 h, the intracellular concentrations of PA and NAD^+^/NADH were determined with Pyruvate Assay Kit and NAD^+^/NADH detection kit (Solarbio® life sciences) according to the manufacturer’s protocols. For western blots analysis, the protein samples were extracted from the treated cells according to the manufacturer’s protocols. The expression of NAMPT and histones were analyzed by ProteinSimple Wes^TM^ Capillary Western Blot analyzer (PS-MK15, ProteinSimple, version 6.0.0).

To verify the roles of NAMPT and NAD^+^ on histones expression, the cells were simultaneously treated with M@HLPC + LA and siNAMPT (or FK866, an NAD^+^ biosynthesis inhibitor^56^) or treated with pcDNA3.1-NAMPT. The expression of H2A and H2B were analyzed by ProteinSimple Wes^TM^ Capillary Western Blot analyzer. To study the effect of MEF2C on NAMPT expression, the cells were simultaneously treated with M@HLPC + LA and siMEF2C or treated with pcDNA3.1-MEF2C. To study the roles of histones on cell proliferation, the cells were simultaneously treated with M@HLPC + LA and pcDNA3.1-H2A/H2B, and then stained with a proliferation marker (Ki67) for flow cytometry analysis. The expression of MEF2C, NAMPT, H2A, and H2B were analyzed by ProteinSimple Wes^TM^ Capillary Western Blot analyzer, CLSM, or flow cytometry. The uncropped scans of the western blotting data were supplied in Supplementary Figs. 14–16 and 18.

For cell cycle analysis, the cells were first synchronized with 1.5 mM hydroxyurea (HU). After receiving the indicated treatments as above, the cells were collected at different time points and fixed with 70% ethanol overnight. Then, the cells were stained with 50 g/mL PI and measured by flow cytometry according to the manufacturer’s protocols. For cell proliferation analysis, the cells were received the indicated treatments as above. After collected, the cells were stained with Ki67 and analyzed by flow cytometry.

To verify the chemiexcited PDT effect, hypoxic U251 cells were cultured in glass-bottom dishes or 24-well plates and treated with PBS, LA (5 mM), M@HLPC, M@HLPC + LA (5 mM), or sodium pyruvate (5 mM, positive control). For intracellular ^1^O_2_ detection, 10 mM DCFH-DA was added. The cells were analyzed by CLSM and flow cytometry. For apoptosis assays, the cells receiving the indicated treatments were detected by flow cytometry after stained with Annexin V-FITC/PI according to the manufacturer’s instructions.

#### Cell viability detection

Hypoxic U251 cells (1 × 10^4^ cells per well) were seeded in 96-well plates and incubated with M@HLPC, M@LPC + LA (5 mM), M@HPC + LA (5 mM), and M@HLP + LA (5 mM), M@HLPC + LA (5 mM) for 24 h. The standard CCK-8 assay was used to evaluate the cell viability after the cells were incubated for another 4 h.

### In vivo synergistic antitumor efficacy of M@HLPC

#### Therapeutic effect and safety analysis

Orthotopic glioma-bearing mice were randomly divided into five groups (6 mice per group) and received five times i.v. injection of 200 μL PBS, M@HLP, M@HPC, M@HLPC, or naked HLPC (all at a dose equivalent of 1.0 mg/kg Ce6 and 1.3 mg/kg LOX per mouse). The growth of the tumors was monitored by the assistance of bioluminescence imaging on days 9, 12, 15, 18, 21, 24, and 27. The body weight change of each mouse was recorded every other day. The survival percent was measured every 2 days, and the experimental endpoint was defined as either death or luciferase signal intensity in bioluminescence imaging higher than 10^8^ a.u. The mice bearing tumor over 10^8^ a.u. received euthanasia, rather than being left to die due to excessive tumor burden. To evaluate the safety of M@HLPC, blood was collected for serum biochemistry. The major organs were collected and sectioned into 10-μm slices. After staining with hematoxylin and eosin (H&E), the slices were examined by an automatic multispectral imaging system.

#### LA, PA, NAMPT, histone, and Ki67 proliferation analysis

The glioma-bearing mice were randomly divided into four groups (3 mice per group) and received i.v. injection of PBS, M@HLP, M@HLP, or M@HLPC (200 μL) on days 9, 12, 15. In this experiment, 5 μL SOSG was intratumorally injected into the tumor to consume the produced ^1^O_2_. On day 16, the tumors were collected and triturated into single cells suspension, the LA and PA concentration was determined by Lactate Assay Kit and Pyruvate Assay Kit according to the supplier’s operating instructions. The ﻿expression of NAMPT and histone (H2A, H2B, and H4) were analyzed using ProteinSimple Wes^TM^ Capillary Western Blot analyzer after the protein samples were extracted from the single cells suspension. The brain of each mouse was collected for Ki67 staining.

#### ^1^O_2_ and apoptosis detection

The glioma-bearing mice were randomly divided into four groups (3 mice per group) and received i.v. injection of PBS, M@HLP, M@HLP, or M@HLPC (200 μL) on days 9, 12, 15. In this experiment, 5 μL (10 mM) DCFH-DA was intratumorally injected into the tumor to capture the produced ^1^O_2_. After 24 h post-injection, the tumor was imaged by a multiphoton laser confocal microscope with an 800 nm excitation. The brain of each mouse was collected for terminal deoxynucleotidyl transferase-mediated dUTP-biotin nick end labeling assay (TUNEL) staining.

### Construction and characterization of hM@HLPC

The reserved part (one part for the construction of PDX model) of the tumor from GBM patient was cut into tiny pieces and triturated into single cells suspension. The cell membranes of the human tumor tissue (hM) were prepared using the same protocol as the aforementioned U251 cells. For construction of hM@HLPC, the HLPC was coextruded with hM. The size and zeta potential of HLPC and hM@HLPC were measured via a ZetaSizer at 25 °C. TEM image of hM@HLPC was recorded on JEOL JEM-1400. To verify hM on the HLPC particles by fluorescence imaging, hM@HLPC was fixed on glass-bottom dishes and incubation with DiO for 1 h. After being washed, the nanoparticles were viewed by ultrahigh-resolution fluorescence microscope. The in vitro BBB-penetrating capacity and synergistic antitumor mechanism of hM@HLPC were performed on GBM patient-derived cells as that of U251 cells.

### In vivo targeting ability of hM@HLPC

After the tumor was determined using an MRI system, PDX mice were divided into two groups (3 mice per group) and injected (i.v.) with HLPC or hM@HLPC (200 μL, Ce6: 1.0 mg/kg). Fluorescence imaging was taken at different time intervals using an IVIS imaging system.

### In vivo antitumor efficacy of hM@HLPC against PDX tumors

After the tumors were determined using an MRI system, PDX mice were divided into three groups (6 mice per group) and received five times i.v. injection of PBS, HLPC, or hM@HLPC (200 μL, Ce6: 1.0 mg/kg). Tumor progression was assessed by MRI at 7 day and 21 day.

### Statistical analysis

All data were presented as mean ± SD. Statistical analysis was performed with GraphPad Prism 8.0.1 software by two-tailed unpaired Student’s *t*-tests, log-rank test, or one-way ANOVA.

### Reporting summary

Further information on research design is available in the Nature Research Reporting Summary linked to this article.

## Supplementary information

Supplementary Information

Reporting Summary

## Data Availability

The main data supporting the results in this study are available within the paper and its Supplementary Information. Source data for the figures in the main text are available at Figshare (10.6084/m9.figshare.19825075.v1). Source data for the figures in the Supplementary Information are available at Figshare (10.6084/m9.figshare.19825096.v1). The RNA-seq has been deposited to NCBI under accession codes PRJNA747130. The mass spectrometry proteomics data has been deposited to the ProteomeXchange Consortium via the iProX partner repository with the dataset identifier PXD030760. The dataset of LDHA/MCT4 expression in glioma patients (including 524 LGG and 167 HGG cases) was freely obtained from TCGA through cBioportal (URL: http://www.cbioportal.org/). Source data are provided with this paper.

## Source data

Source Data
