# Supplementary material for: Engineered biomimetic nanoparticles achieve targeted delivery and efficient metabolism-based synergistic therapy against glioblastoma
Source: Nat Commun. 2022 Jul 21;13:4214. doi: 10.1038/s41467-022-31799-y (PMC9304377; doi:10.1038/s41467-022-31799-y)
Supplement: Supplementary file 1 — Supplementary Information [file 41467_2022_31799_MOESM1_ESM.pdf]

Engineered biomimetic nanoparticles achieve targeted delivery and efficient metabolism-based synergistic therapy against glioblastoma

Guihong Lu[1, 2], Xiaojun Wang[2], Feng Li[2, 3], Shuang Wang[2], Jiawei Zhao[2], Jinyi Wang[1], Jing Liu[1], Chengliang Lyu[2], Peng Ye[2], Hui Tan[1, 4*], Weiping Li[1*], Guanghui Ma[2, 3*], and Wei Wei[2, 3*]

[1] Department of Neurosurgery, Health Science Center, The First Affiliated Hospital of Shenzhen University, Shenzhen Second People's Hospital, Shenzhen, 518035, P. R. China.

[2] State Key Laboratory of Biochemical Engineering, Institute of Process Engineering, Chinese Academy of Sciences, Beijing, 100190, P. R. China.

[3] School of Chemical Engineering, University of Chinese Academy of Sciences, Beijing 100049, P. R. China.

[4] Pneumology Department, Shenzhen Children's Hospital, Shenzhen, 518026, P. R. China.

*Correspondence author: Wei Wei (weiwei@ipe.ac.cn), Guanghui Ma (ghma@ipe.ac.cn), Weiping Li (wpli@szu.edu.cn), and Hui Tan (huitan@email.szu.edu.cn)

Content

Supplemenetary Fig. 1 Correlation analysis between representative LA metabolism-related indicators (LDHA/MCT4) and proliferation marker (Ki67) in patients by 2D graph of Fig. 2b in manuscript

Supplemenetary Fig. 2 Condition optimization for HLPC construction

Supplemenetary Fig. 3 Standard curves of Hb, CPPO, and Ce6

Supplemenetary Fig. 4 The absorption (Ab) properties of free Ce6 and M@HLPC

Supplemenetary Fig. 5 Production of $^1O_2$ measured by ABDA fluorescence (FL) intensity in hypoxic PBS

Supplemenetary Fig. 6 Flow cytometry gating strategy for Fig. 4a, b

Supplemenetary Fig. 7 Effect of proteins on homotypic uptake

Supplemenetary Fig. 8 In vivo evaluation for the glioma-targeting of M(-)@HLPC in orthotopic U251-luc glioma-bearing mice

Supplemenetary Fig. 9 Investigation of BBB crossing mechanisms in in vitro BBB model

Supplemenetary Fig. 10 In vivo evaluation for the glioma-targeting of M@HLPC in orthotopic GL261-luc glioma-bearing mice

Supplemenetary Fig. 11 Representative ex vivo fluorescent images and corresponding quantitative fluorescence analysis of glioma tumors and major organs dissected from large-size glioma-bearing mice at 24 h after i.v. injection with HLPC or M@HLPC

Supplemenetary Fig. 12 Proposed mechanism for histones repression by M@HLPC

Supplemenetary Fig. 13 In vitro evaluation of the therapeutic effects of exogenous PA against U251 cells

Supplemenetary Fig. 14 The full scans of western blotting data in Fig. 6d

Supplemenetary Fig. 15 Effect of MEF2C on NAMPT expression

Supplemenetary Fig. 16 Effect of NAMPT and NAD^+ on histones expression

Supplemenetary Fig. 17 Cell cycle progression with different treatments, determined by flow cytometry

Supplemenetary Fig. 18 Effect of histones on cell proliferation

Supplemenetary Fig. 19 In vitro evaluation of the synergistic therapeutic effects of M@HLPC against hypoxic U251 cells

Supplemenetary Fig. 20 Relative cell viability of U251 cells after treatments with M@HLP, M@HPC, or M@HLPC at various concentrations

Supplemenetary Fig. 21 In vivo evaluation of the therapeutic effect of HLPC in U251-luc tumor-bearing mice

Supplemenetary Fig. 22 Side effect evaluation via detection of hematological parameter in U251-luc tumor-bearing mice

Supplemenetary Fig. 23 H&E staining of heart, liver, spleen, lung, and kidney slices after treatment with M@HLPC in U251-luc tumor-bearing mice

Supplemenetary Fig. 24 The full scans of Fig. 7g

Supplemenetary Fig. 25 Representative cell proliferation (indicated by Ki67) and apoptosis (indicated by TUNEL) analysis of U251-luc tumor tissue

Supplemenetary Fig. 26 In vitro BBB penetration ability of hM@HLPC

Supplemenetary Fig. 27 In vitro evaluation of the synergistic therapeutic effects of hM@HLPC against GBM patient-derived cells

Supplemenetary Fig. 28 Representative ex vivo fluorescent images and corresponding quantitative fluorescence analysis of glioma tumors and major organs dissected from PDX models at 24 h after i.v. injection with HLPC or hM@HLPC

Supplemenetary Table 1 List of cell membrane proteins selected basing on their function

Supplemenetary Table 2 List of antibodies used in this study

Supplemenetary Table 3 List of siRNA used in this study

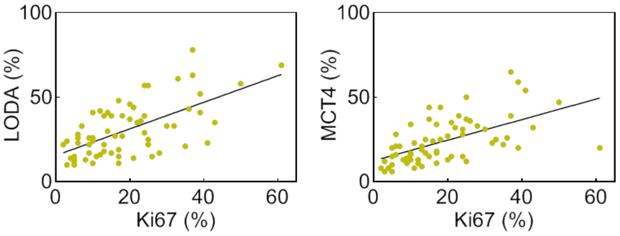

Supplemenetary Fig. 1 Correlation analysis between representative LA metabolism-related indicators (LDHA/MCT4) and proliferation marker (Ki67) in patients by 2D graph of Fig. 2b in manuscript. Positive correlations between LA metabolism-related indicators and Ki67 were observed, indicating the important role of LA in glioma development.

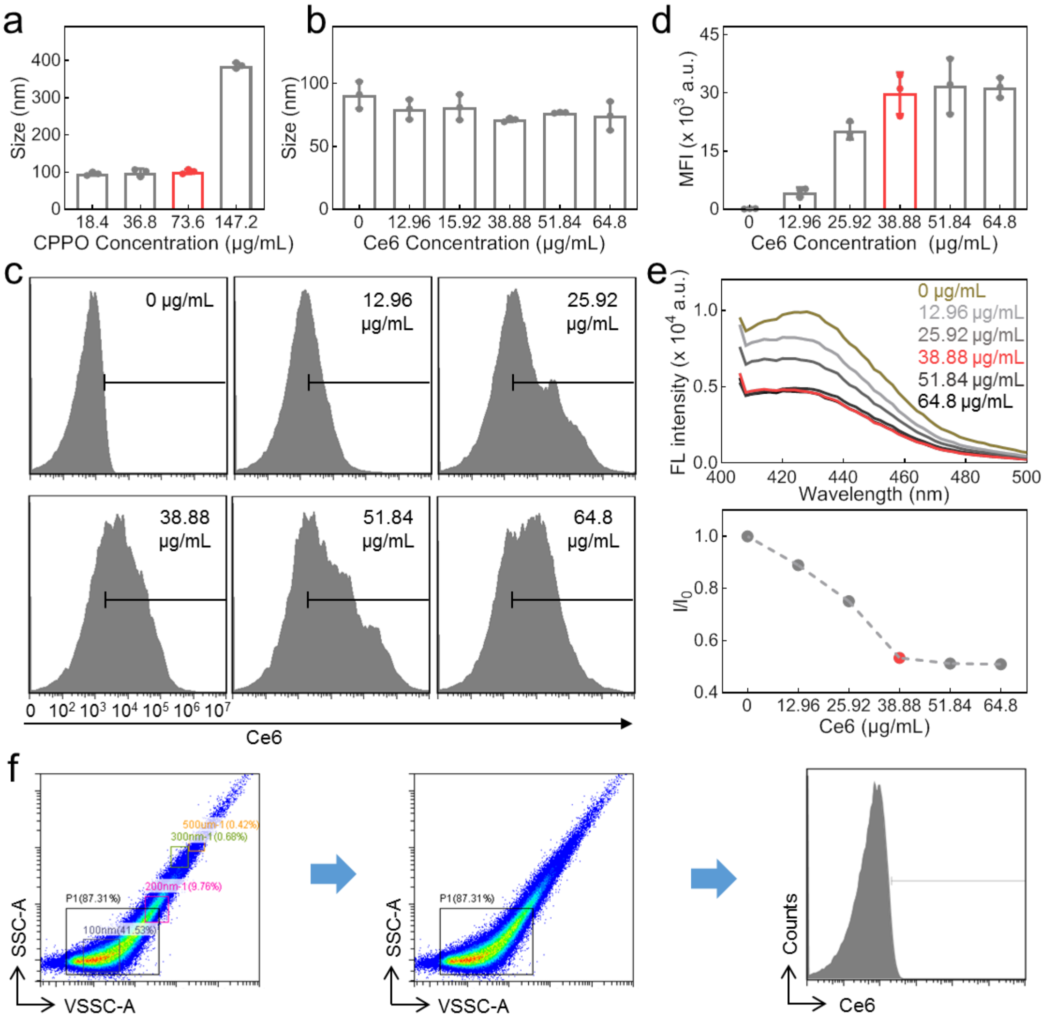

Supplemenetary Fig. 2 Condition optimization for HLPC construction. (a) Size distribution of HLPC at different concentrations of CPPO. The size of HLPC increased to ~ 400 nm when the CPPO concentration was 147.2 μg/mL. Thus, the incorporation of CPPO was optimized at the concentration of 73.6 μg/mL. (b) Size distribution of HLPC at different concentrations of Ce6. The size of HLPC was almost unchanged in all treated Ce6 concentrations. (c) Flow cytometry analysis of HLPC at different concentrations of Ce6. (d) The corresponding mean fluorescence intensities (MFI) in (c), which initially increased from 0 to 38.88 μg/mL, and saturated when 38.88 μg/mL Ce6 was used. (e) Production of $^1O_2$ of HLPC at different concentrations of Ce6 using the 9,10-Anthracenediyl-bis(methylene) dimalonic acid (ABDA) probe. (f) Flow cytometry gating strategy for c. The $^1O_2$ amount increased with increasing Ce6 concentrations and reached maximum value at

38.88 μg/mL of Ce6. Thus, the incorporation of Ce6 was optimized at the concentration of 38.88 μg/mL. Data in a, b, and d were presented as the mean ± SD, n = 3 independent samples.

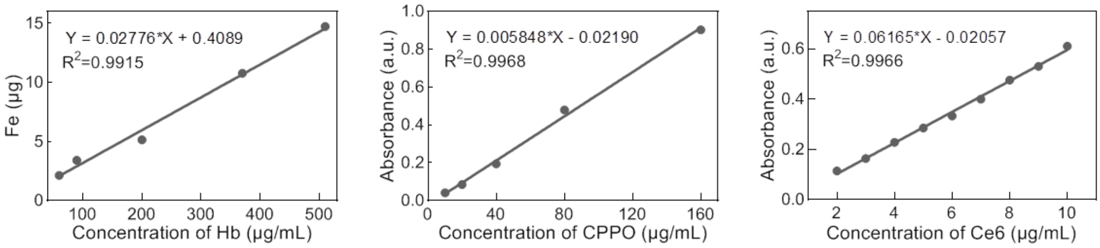

Supplemenetary Fig. 3 Standard curves of Hb, CPPO, and Ce6. The concentration of total protein (Hb and LOX) was measured by the BCA assay. The LOX content in HLPC was determined by subtracting Hb (analyzed using ICP-OES) from the total protein. The CPPO and Ce6 in HLPC were verified by UV/vis absorption spectra. After calculating, the content of LOX, CPPO, and Ce6 were determined to be 6.7 ± 0.9, 9.4 ± 0.7 and 5.1 ± 0.4 μg per 100 μg NPs, respectively.

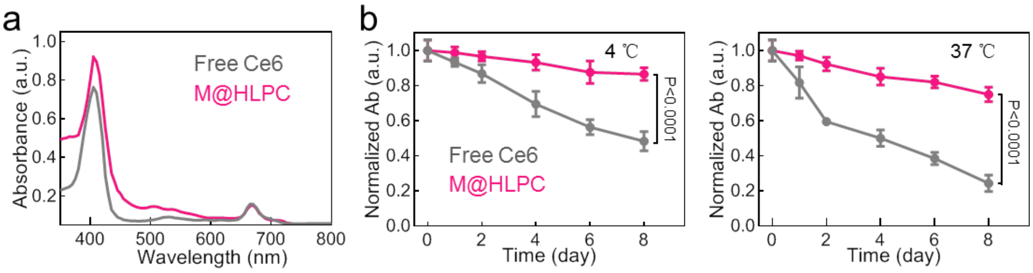

Supplemenetary Fig. 4 The absorption (Ab) properties of free Ce6 and M@HLPC. (a) UV/vis Ab spectra of free Ce6 and M@HLPC. The Ab spectra of Ce6 were almost unchanged in M@HLPC, indicating that the Ab properties of Ce6 were not influenced by NPs encapsulating. (b) The Ab stability of free Ce6 and M@HLPC for 8 days at 4 ℃ (left) and 37 ℃ (right). Encapsulating Ce6 in NPs could significantly improve the Ab stability of Ce6, ensuring its stable activity for $^1O_2$ generation during the chemiexcited PDT. Data in b were presented as the mean ± SD, n = 3 independent samples. P values were calculated by using two-tailed unpaired Student's *t*-test.

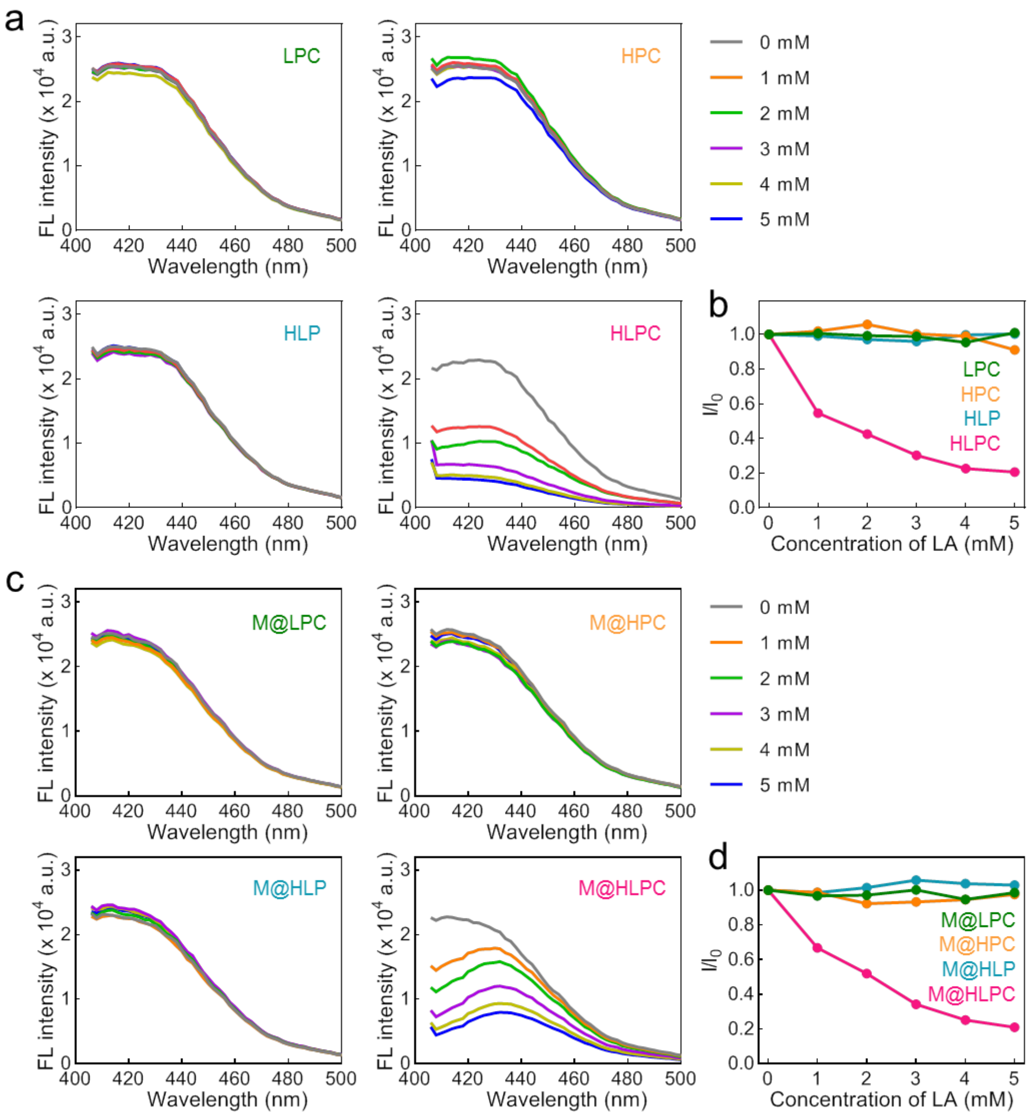

Supplemenetary Fig. 5 Production of $^1O_2$ measured by ABDA fluorescence (FL) intensity in hypoxic PBS. (a) FL spectra of ABDA incubated with LPC, HPC, HLP, or HLPC at different concentrations of LA. (b) Normalized FL intensity of ABDA at 410 nm (upon excitation at 378 nm) as a function of LA concentrations. Only HLPC samples exhibited decreased ABDA signals, and the signal decrement was LA concentration-dependent. (c) FL spectra of ABDA incubated with M@LPC, M@HPC, M@HLP, or M@HLPC at different concentrations of LA. (d) Normalized FL intensity of ABDA at 410 nm as a function of LA concentrations. Only M@HLPC samples exhibited decreased ABDA signals, and the signal decrement was LA concentration-dependent, indicating M@HLPC could respond to LA and release H_2O_2 for $^1O_2$ generation through the CPPO-excited Ce6.

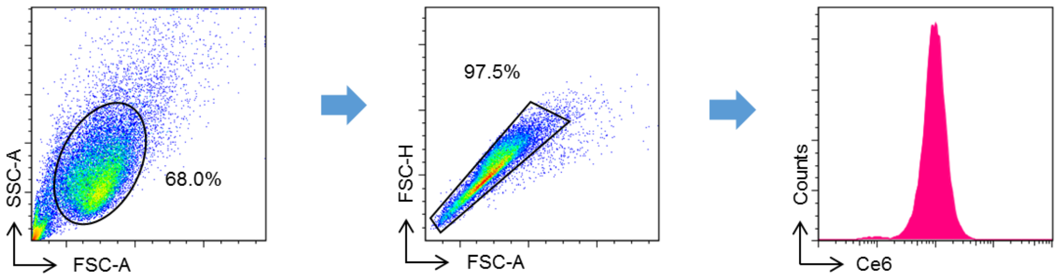

Supplemenetary Fig. 6 Flow cytometry gating strategy for Fig. 4a, b. The single cells were identified by FSC and SSC.

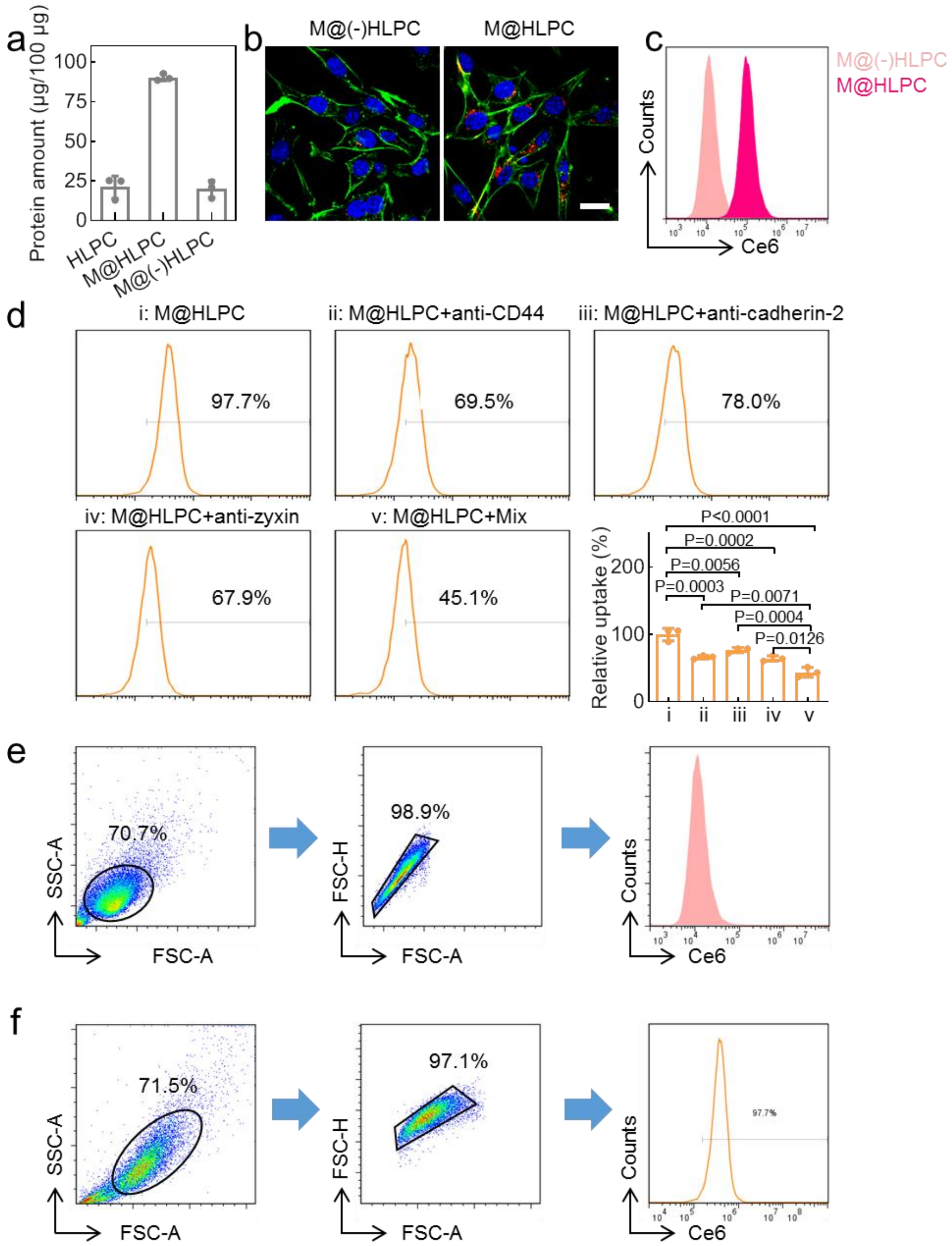

Supplemenetary Fig. 7 Effect of proteins on homotypic uptake. (a) Protein content in HLPC, M@HLPC, and M(-)@HLPC (de-proteinated cell membrane-coated HLPC). Compared to the M@HLPC, M(-)@HLPC showed a significant reduction of protein amount. Meanwhile, the protein amount of M(-)@HLPC was much similar to that of HLPC. These two aspects together voted the successful removal of proteins on the coated cell membrane. (b) Representative CLSM images of U251 incubated with M@HLPC or M(-)@HLPC. Red: NPs; blue: Hoechst 33342-labeled nuclei; green:

rhodamine phalloidin-labeled U251 cells. Scale bar: 25 μm. (c) Flow cytometry analysis of U251 incubated with M@HLPC or M(-)@HLPC. Lower uptake of M(-)@HLPC by U251 cells were observed, indicating the role of the protein components on the coated membrane for facilitating the M@HLPC homotypic uptake. (d) Uptake efficiency of M@HLPC, M@HLPC+anti-CD44, M@HLPC+anti-cadherin-2, M@HLPC+anti-zyxin, and M@HLPC+Mix (combination of anti-CD44, anti-cadherin-2, and anti-zyxin) by the parent U251 cells. The uptake could be inhibited after pretreating M@HLPC with anti-CD44, anti-cadherin-2, or anti-zyxin. Moreover, combined utilization of these three types of antibodies further magnified the uptake inhibition. These representative results thus indicated M@HLPC' homotypic targeting highly associated to the proteins on the coated cell membrane. (e) Flow cytometry gating strategy for c. (f) Flow cytometry gating strategy for d. Data in a and d were presented as the mean ± SD, n = 3 independent samples. The experiments in b were repeated independently three times with similar results. P values were calculated by using one-way ANOVA.

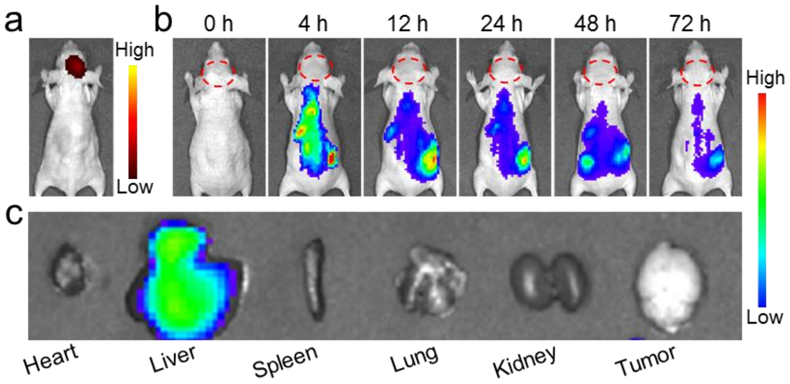

Supplemenetary Fig. 8 In vivo evaluation for the glioma-targeting of M(-)@HLPC in orthotopic U251-luc glioma-bearing mice. (a) Representative bioluminescence image of small-size glioma-bearing mice at day 9 post tumor cell inoculation. (b) In vivo distributions of M(-)@HLPC in small-size U251-luc glioma-bearing Balb/c nude mice at different time points post i.v. injection. (c) Representative ex vivo fluorescence images of major organs dissected from glioma-bearing mice at 24 h after i.v. injection with M(-)@HLPC. The fluorescence signals originate from Ce6 inside the formed NPs. There was no obvious Ce6 signal in the brains of the M(-)HLPC-treated mice, demonstrating the de-proteinated M(-)HLPC could not traverse the slightly disrupted BBB and accumulate in gliomas at an early stage. Images were representative of three independent mice.

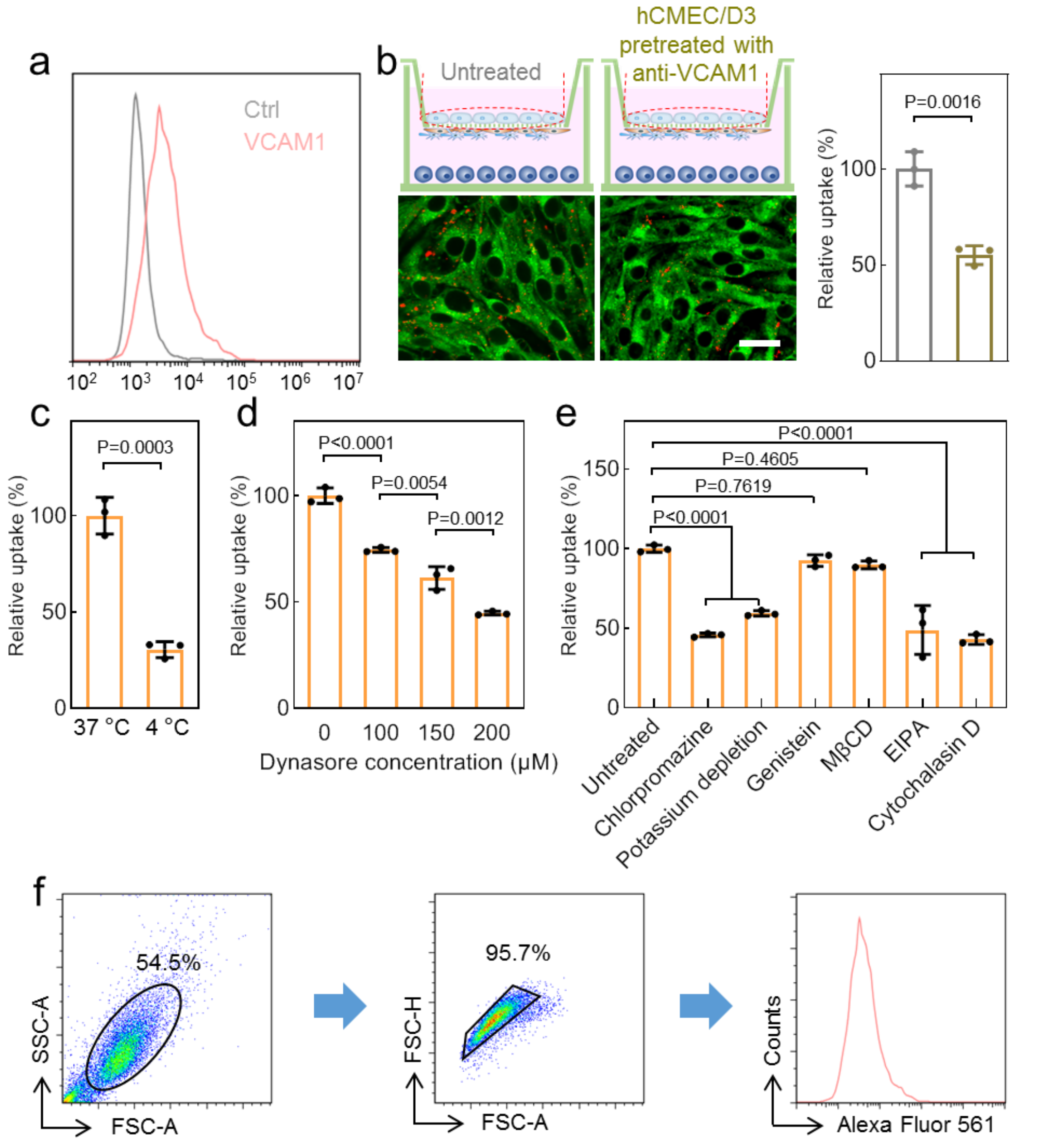

Supplemenetary Fig. 9 Investigation of BBB crossing mechanisms in in vitro BBB model. (a) Flow cytometry analysis of VCAM1 expression on hCMEC/D3 cells. (b) CLSM images and flow cytometry analysis of hCMEC/D3 and anti-VCAM1-blocked hCMEC/D3 in the upper chamber incubated with M@HLPC. Red: NPs (Ce6 signal); green: DIO-labeled hCMEC/D3. Scale bar: 25 μm. The uptake in the untreated group was normalized to 100%. The uptake of M@HLPC could be inhibited after pretreating hCMEC/D3 with anti-VCAM1. (c) Relative uptake analysis of M@HLPC by hCMEC/D3 in the upper chamber at 4 ℃ or 37 ℃. The uptake at 37 ℃ was normalized to 100%. The uptake of M@HLPC by hCMEC/D3 at 4 ℃ was reduced to 35.3%, compared to that at 37 ℃. (d) Relative uptake analysis of

M@HLPC by hCMEC/D3 pretreated with different concentration of Dynasore (an inhibitor of endocytosis). The uptake of M@HLPC by hCMEC/D3 cells gradually decreased with the increased dose of Dynasore. (e) Relative uptake analysis of M@HLPC by hCMEC/D3 cells pretreated by different endocytosis inhibitors, including chlorpromazine and potassium depletion for inhibition of clathrin-mediated endocytosis pathway, genistein and MβCD for inhibition of caveola-mediated endocytosis pathway, 5-(N-ethyl-N-isopropyl) amiloride (EIPA) and cytochalasin D for inhibition of micropinocytosis. Treatment with chlorpromazine, potassium depletion, EIPA, or cytochalasin D resulted in the significant inhibition of M@HLPC uptake, suggesting the involvement of the clathrin-dependent pathway and macropinocytosis for the transcytosis of M@HLPC across the BBB. (f) Flow cytometry gating strategy for a. Quantitative data in b-e were presented as the mean ± SD, n = 3 independent samples. The experiments in b were repeated independently three times with similar results. P values were calculated by using two-tailed unpaired Student's *t*-test (b, c) or one-way ANOVA (d, e).

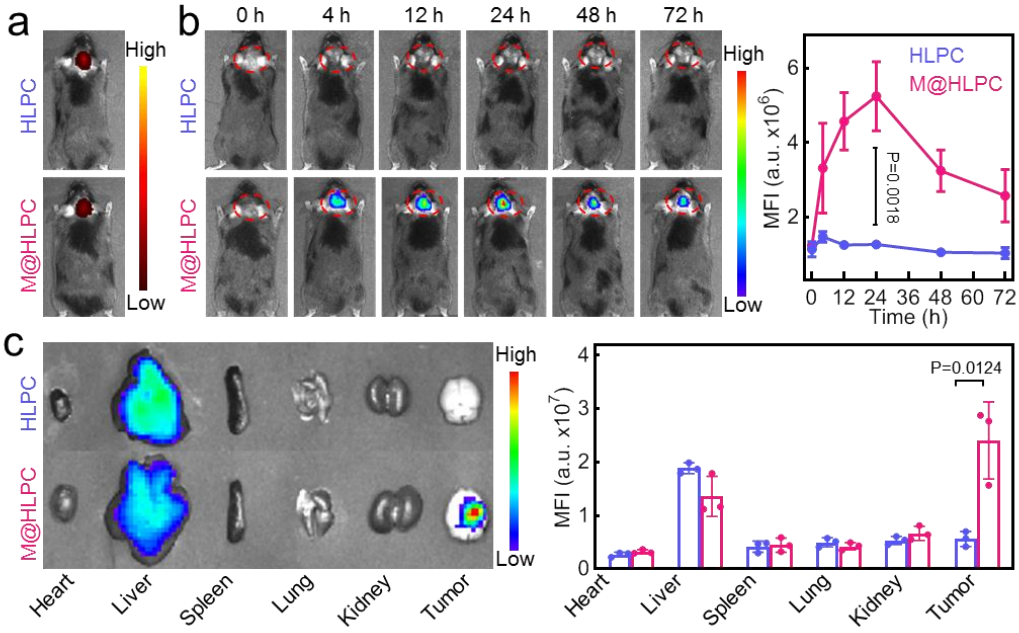

Supplemenetary Fig. 10 In vivo evaluation for the glioma-targeting of M@HLPC in orthotopic GL261-luc glioma-bearing mice. (a) Representative bioluminescence images of small-size glioma-bearing mice at day 9 post tumor cell inoculation. The mice were randomly divided into two groups and i.v. injected with HLPC or M@HLPC for the subsequent distribution analysis. (b) In vivo distributions and the signal profiles of HLPC or M@HLPC in small-size GL261-luc glioma-bearing C57BL/6 mice at different time points post i.v. injection. (c) Representative ex vivo fluorescence images and corresponding quantitative fluorescence analysis of major organs and brain tumors dissected from glioma-bearing mice at 24 h after i.v. injection with HLPC or M@HLPC. The Ce6 signal was evident in the brains of the M@HLPC-treated mice rather than the HLPC-treated mice. Further ex vivo imaging of the brains and other main organs also showed a unique signal in the brain tumor region of the M@HLPC-treated mice. Quantitative data in b and c were presented as the mean ± SD, n=1 experiment, n=3 mice per group. Images were representative of three independent mice. P values were calculated by using two-tailed unpaired Student's *t*-test.

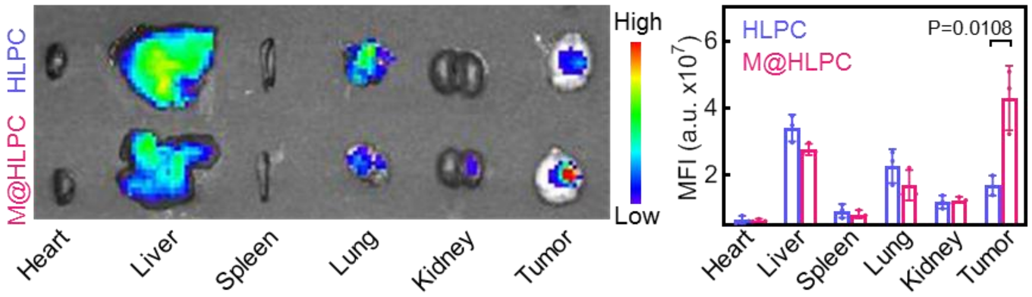

Supplemenetary Fig. 11 Representative ex vivo fluorescent images and corresponding quantitative fluorescence analysis of glioma tumors and major organs dissected from large-size glioma-bearing mice at 24 h after i.v. injection with HLPC or M@HLPC. M@HLPC showed much superior accumulation compared to that of HLPC in the glioma tumor area, demonstrating the superior BBB-penetration and specific targeting recognition ability to homologous tumors sourced from M camouflage. Quantitative data were presented as the mean ± SD, n=1 experiment, n=3 mice per group. Images were representative of three independent mice. P value was calculated by using two-tailed unpaired Student's *t*-test.

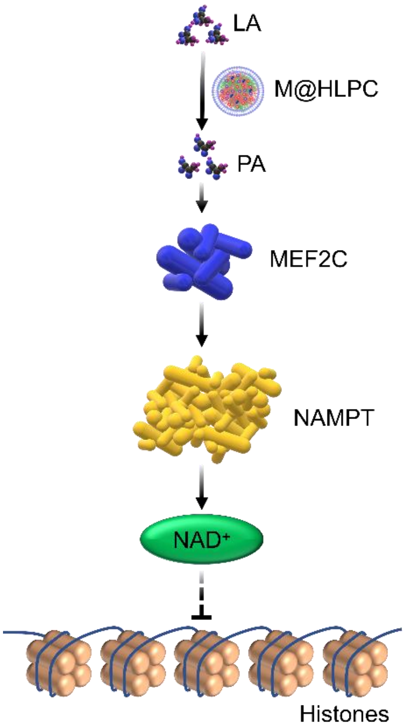

Supplemenetary Fig. 12 Proposed mechanism for histones repression by M@HLPC. LOX in the M@HLPC firstly converted LA into PA. Subsequently, PA upregulated the expression of NAMPT via MEF2C. This elevated NAD^+ and finally reduced histones (such as H2A, H2B, and H4) expression.

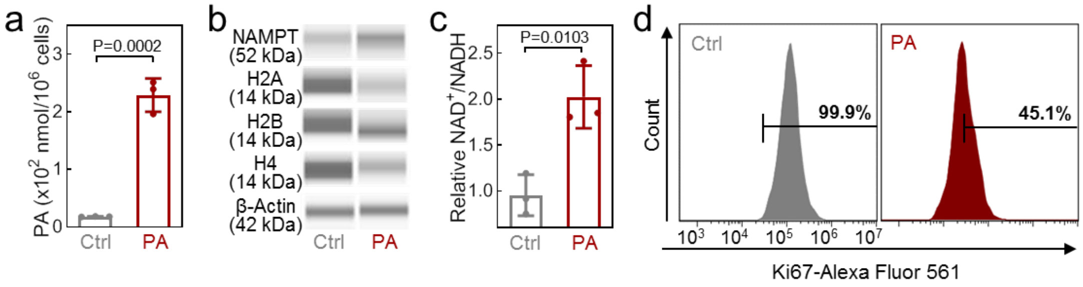

Supplemenetary Fig. 13 In vitro evaluation of the therapeutic effects of exogenous PA against U251 cells. (a) Quantitative analysis of the intracellular PA concentration in U251 cells treated with 5 mM sodium pyruvate. (b) Intracellular NAMPT and histone protein (H2A, H2B, and H4) levels in U251 cells analyzed by ProteinSimple Wes^{TM} Capillary Western Blot analyzer. (c) Analysis of the NAD$^+$/NADH ratio in U251 cells by NAD$^+$/NADH Assay Kit. (d) Proliferation (indicated by Ki67) analysis of U251 cells subjected to 5 mM sodium pyruvate. Data in a and c were presented as the mean ± SD, n = 3 independent samples. P values were calculated by using two-tailed unpaired Student's *t*-test.

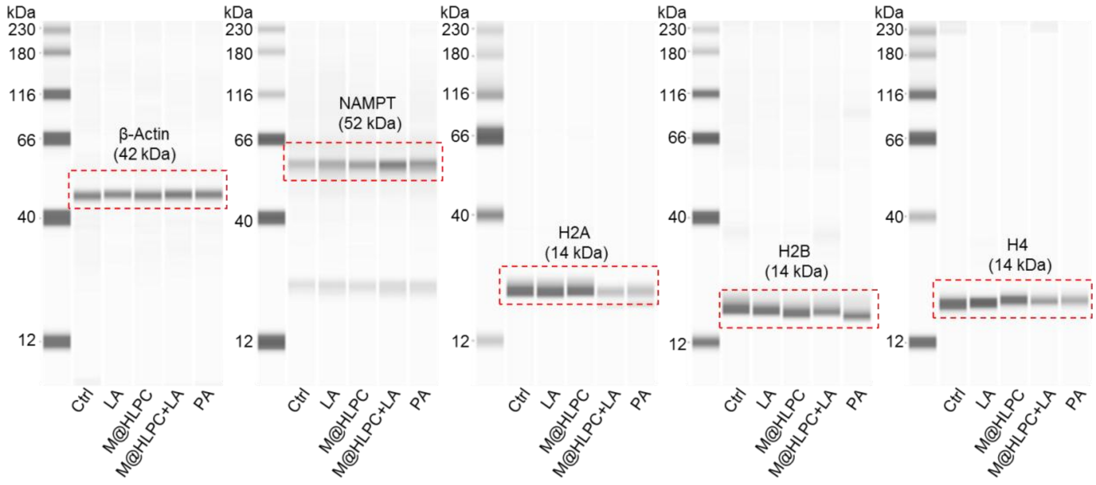

Supplemenetary Fig. 14 The full scans of western blotting data in Fig. 6d. Intracellular MEF2C, NAMPT, and histones (H2A, H2B, H4) levels in U251 cells analyzed by using ProteinSimple Wes^{TM} Capillary Western Blot analyzer. β-Actin was used as housekeeping protein normalizing the levels of protein. These data were derived from the same batch experiment.

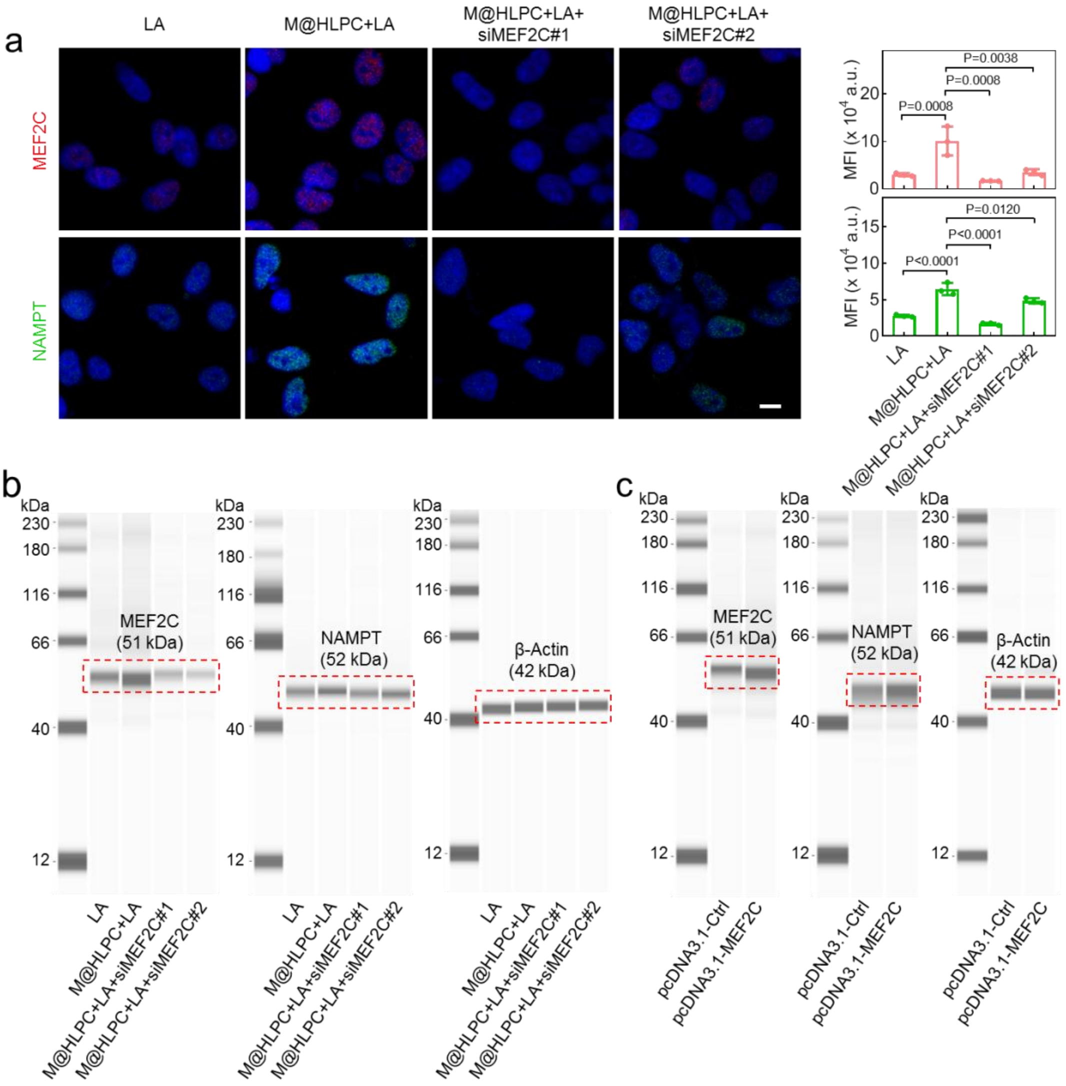

Supplemenetary Fig. 15 Effect of MEF2C on NAMPT expression. Exogenous LA was added to enhance the LA level in the cultured cells. (a) MEF2C and NAMPT levels in cells analyzed by using CLSM and flow cytometry. Blue: nuclei; red: MEF2C; green: NAMPT. Scale bar: 25 μm. (b) MEF2C and NAMPT levels in cells analyzed by using ProteinSimple Wes™ Capillary Western Blot analyzer. Treatment with M@HLPC increased the expression of MEF2C as well as NAMPT in U251 cells. However, when these cells were simultaneously treated with MEF2C siRNA (siMEF2C#1 or siMEF2C#2), the level of NAMPT approximately returned to normal, indicating the upstream regulation of MEF2C on NAMPT. (c) Overexpression of MEF2C with pcDNA3.1-MEF2C upregulated the down-streamed NAMPT. These data were derived from the same batch experiment. Quantitative data in a were presented as the mean ± SD, n = 3 independent samples. The experiments in a were

repeated independently three times with similar results. P values were calculated by using one-way ANOVA.

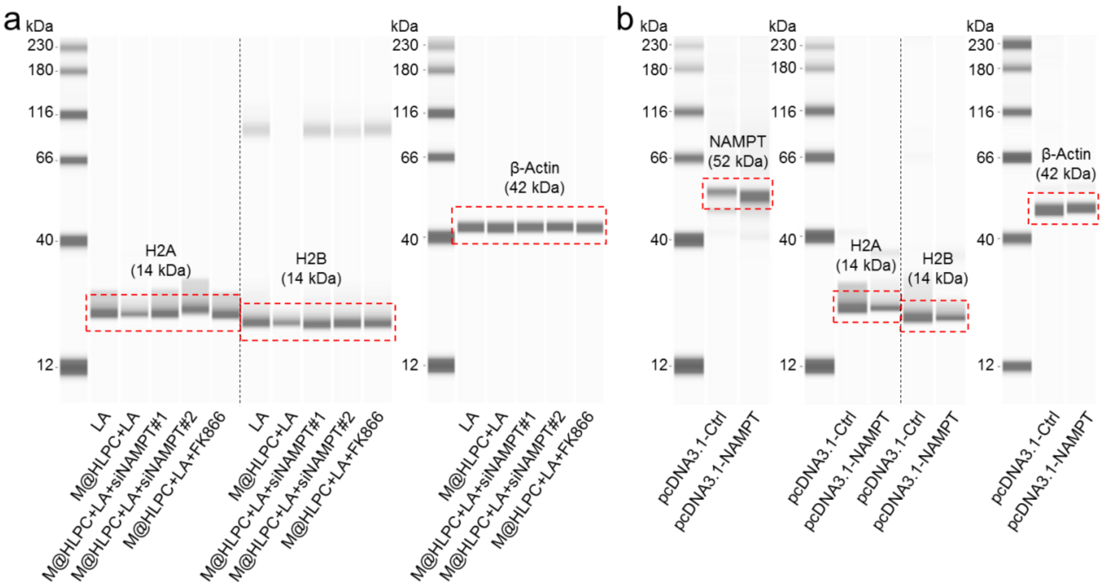

Supplemenetary Fig. 16 Effect of NAMPT and NAD$^+$ on histones expression. Exogenous LA was added to enhance the LA level in the cultured cells. (a) Histones (H2A and H2B) levels in cells analyzed by using ProteinSimple Wes^{TM} Capillary Western Blot analyzer. Treatment with M@HLPC reduced the expression of histones in cells. However, when these cells were simultaneously treated with NAMPT siRNA (siNAMPT#1 or siNAMPT#2) or FK866 (an NAD$^+$ biosynthesis inhibitor), the level of histones approximately returned to normal, indicating the contribution of NAMPT and NAD$^+$ for the histones reduction. (b) Overexpression of NAMPT with pcDNA3.1-NAMPT resulted in the reduction of histones expression. These data were derived from the same batch experiment.

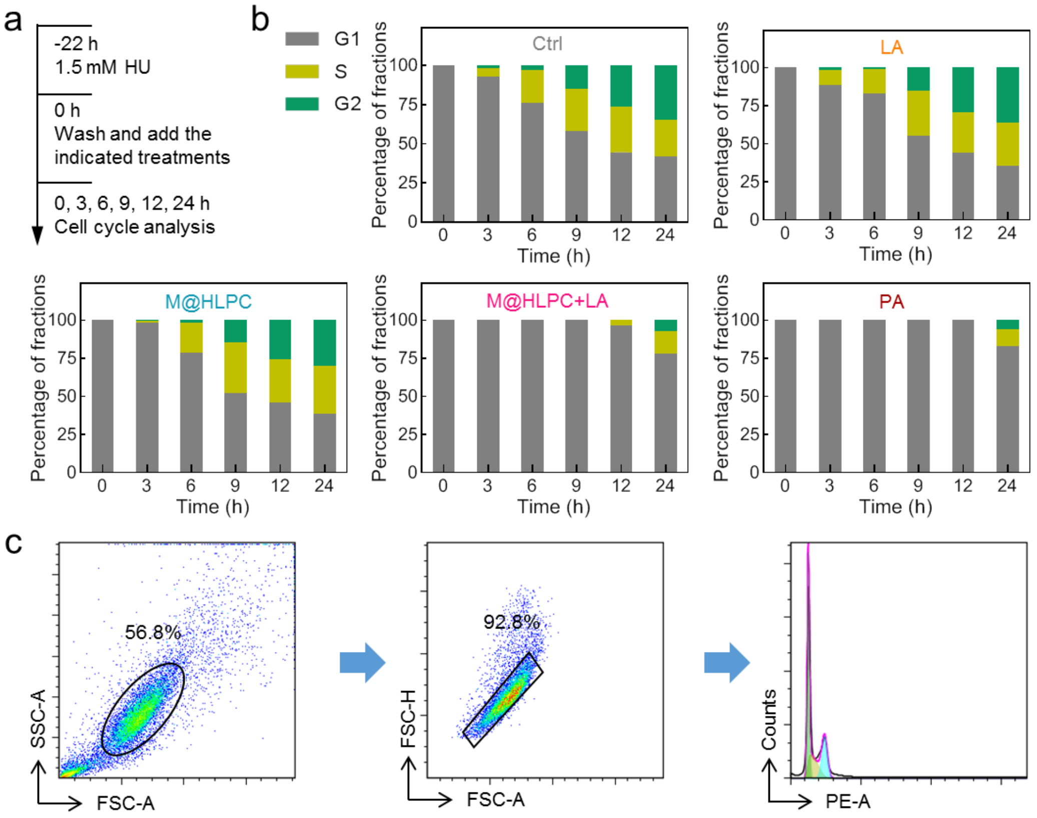

Supplemenetary Fig. 17 Cell cycle progression with different treatments, determined by flow cytometry. G1: G1 phase; S: S phase; G2: G2 phase. (a) Schematic illustration of the experimental design for cell cycle progression. Cells were synchronized in 1.5 mM HU for 22 h. After washing, cells were subjected to the indicated treatments and harvested at different time points for flow cytometry analysis. (b) Cell cycle progression was determined by flow cytometry. (c) Flow cytometry gating strategy for b and Fig. 6f. The results showed that the normal progression to the S phase in the untreated Ctrl, LA, and M@HLPC groups. In contrast, progression to the S phase was delayed in cells treated with M@HLPC+LA and exogenous PA.

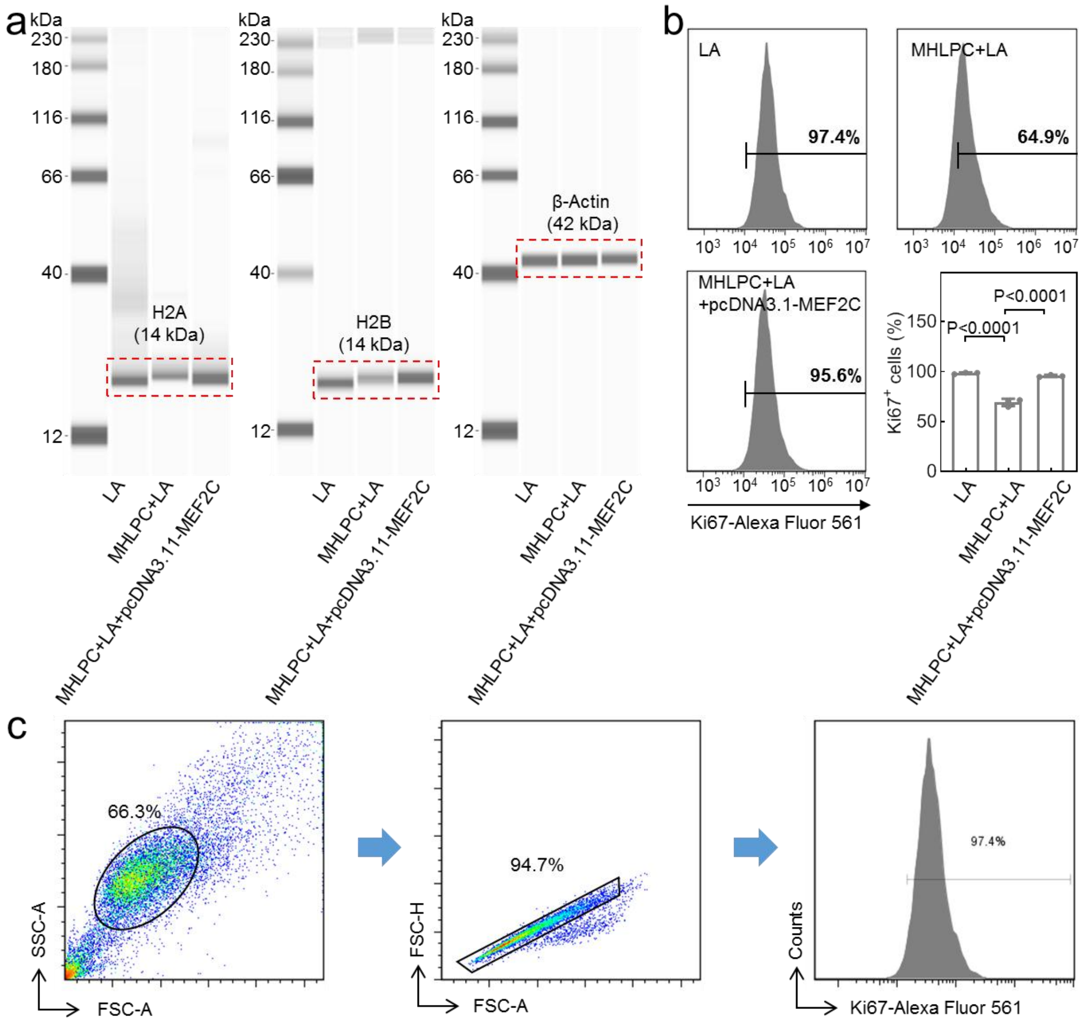

Supplemenetary Fig. 18 Effect of histones on cell proliferation. (a) Overexpression of histone H2A and H2B with pcDNA3.1-H2A/H2B. (b) Effect of M@HLPC+LA on proliferation of cells that overexpressed histones H2A and H2B. (c) Flow cytometry gating strategy for b. Quantitative data in b were presented as the mean ± SD, n = 3 independent samples. P values were calculated by using one-way ANOVA.

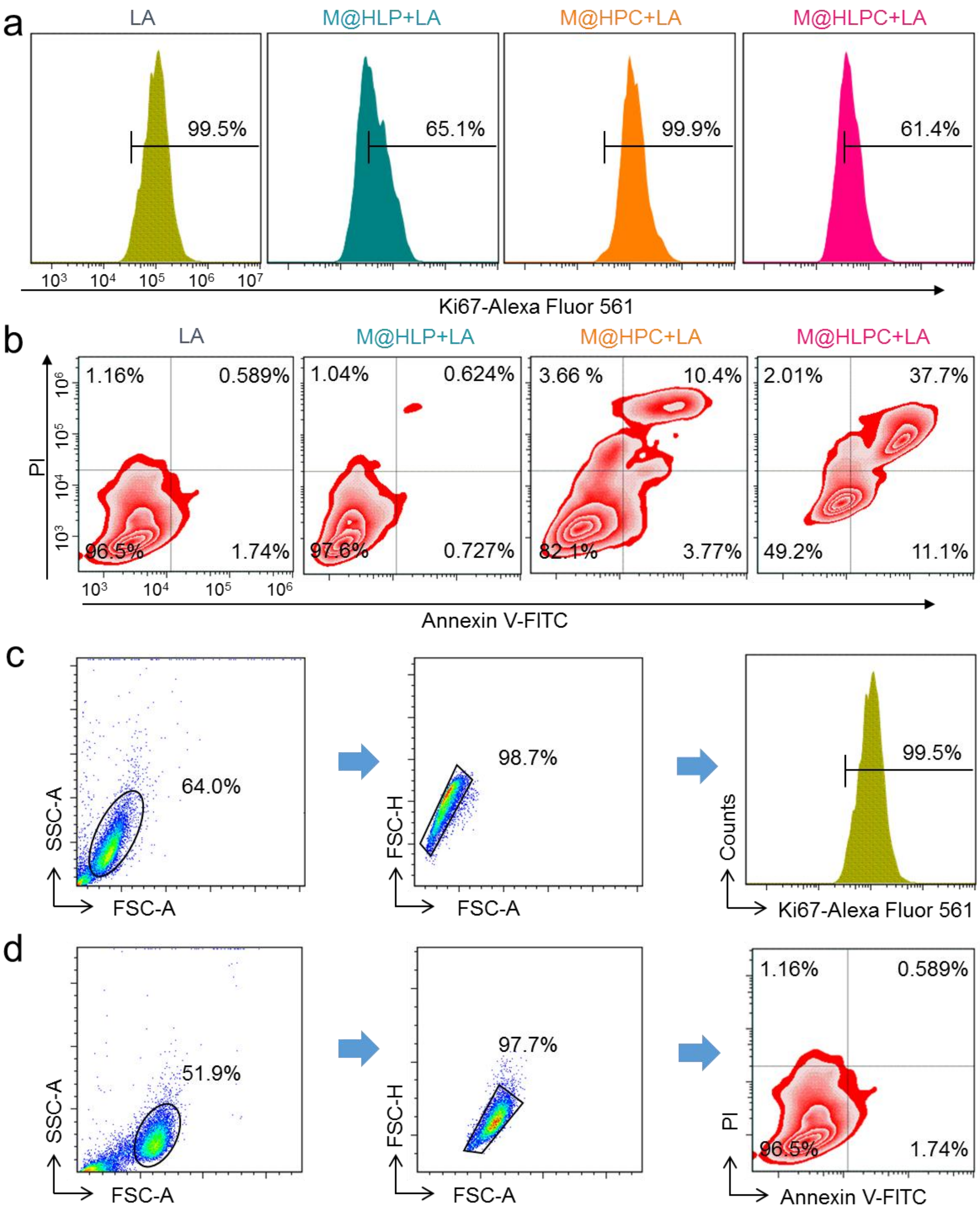

Supplemenetary Fig. 19 In vitro evaluation of the synergistic therapeutic effects of M@HLPC against hypoxic U251 cells. (a) Proliferation (indicated by Ki67) analysis of U251 cells subjected to the indicated formulations with 5 mM LA. Compared to M@HPC+LA group, decreased overall proliferation rates were observed in M@HLP+LA and M@HLPC+LA groups, indicating LOX-catalyzed LA catabolism (LA metabolic therapy) affected cell proliferation. (b) Flow cytometry analysis of U251 cell apoptosis induced by the indicated formulations with 5 mM LA. (c) Flow cytometry gating strategy for a, Supplemenetary Fig. 13d, and Fig. 6g. (d)

Flow cytometry gating strategy for b and Fig. 6i. No apoptosis was detected in M@HLP+LA group, indicating LOX-catalyzed LA catabolism (metabolic therapy) alone had no remarkable effect on cell death. As for the M@HPC+LA group, bits of apoptosis were detected due to the inherent H_2O_2 in cultured cells, indicating M@HPC could respond to the H_2O_2 inside tumor to produce $^1O_2$ for chemiexcited PDT. Notably, much more apoptosis cells were detected in M@HLPC+LA group, supporting the in situ released H_2O_2 during LOX-catalyzed LA catabolism could increase the production of $^1O_2$, and thus induced the highest degree of apoptosis.

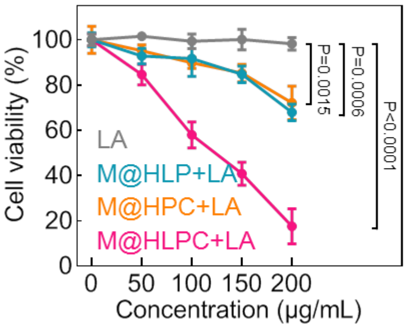

Supplemenetary Fig. 20 Relative cell viability of U251 cells after treatments with M@HLP, M@HPC, or M@HLPC at various concentrations. Although the decrease of cell viability for M@HPC, M@HLP and M@HLPC were concentration-dependent, the decrease for M@HLPC was more pronounced than that for M@HPC and M@HLP. These results indicated that metabolic therapy and chemiexcited PDT could coordinate and enhance the killing effect of M@HLPC on U251 tumor cells. Data were presented as the mean ± SD, n = 3 independent samples. P values were calculated by using one-way ANOVA.

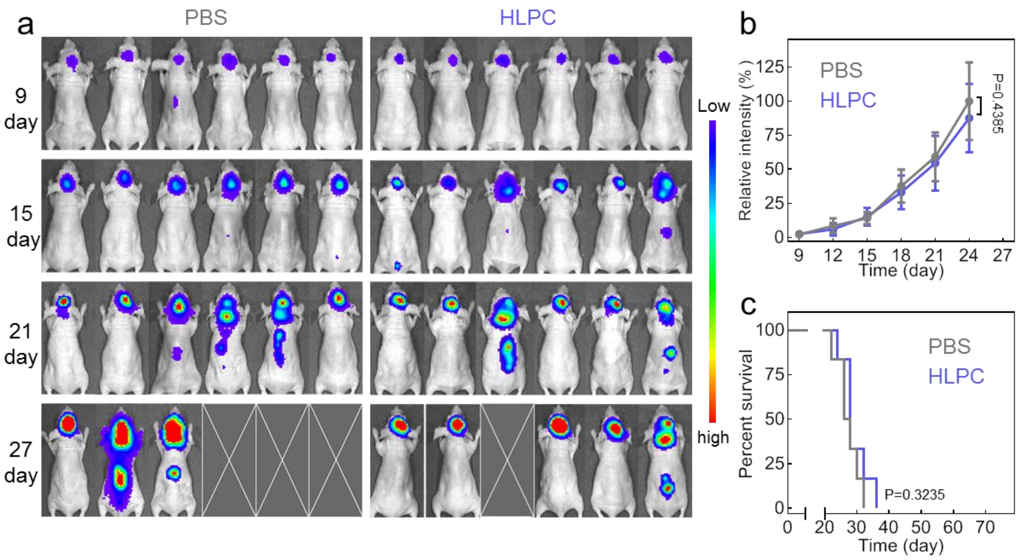

Supplemenetary Fig. 21 In vivo evaluation of the therapeutic effect of HLPC in U251-luc tumor-bearing mice. (a) Bioluminescence images of U251-luc cells in glioma-bearing Balb/c nude mice receiving different treatments at the indicated time points. The blank area indicated that the corresponding mouse had died. (b) Quantification of the bioluminescence signal intensity from bioluminescence images on days 9, 12, 15, 18, 21, and 24. (c) Survival curves of the glioma-bearing mice receiving different treatments. Data in b were presented as the mean ± SD, n=1 experiment, n=6 mice per group. P values were calculated by using two-tailed unpaired Student's *t*-test (b) or Log-rank tests (c).

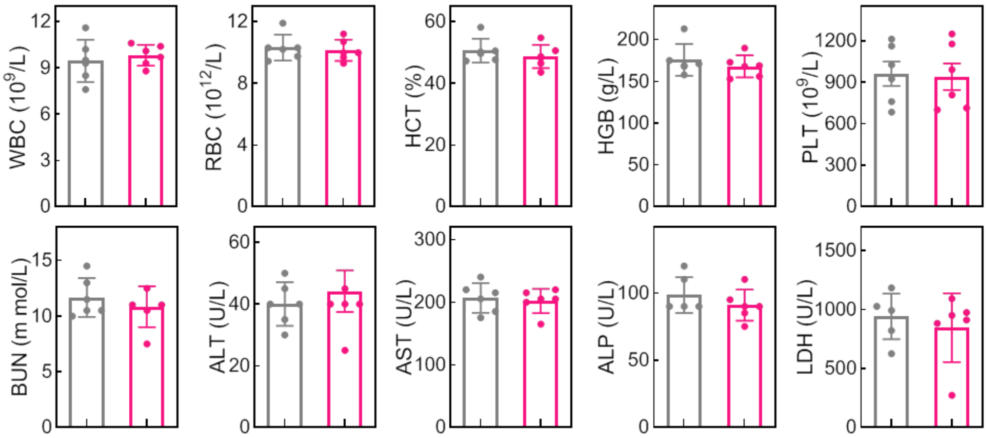

Supplemenetary Fig. 22 Side effect evaluation via detection of hematological parameter in U251-luc tumor-bearing mice (Ctrl: healthy mice without tumor and M@HLPC treatment). The white blood cell (WBC), red blood cell (RBC), haematocrit (HCT), hemoglobin (HGB), platelet (PLT), blood urea nitrogen (BUN), aspartate alanine aminotransferase (ALT), aminotransferase (AST), alkaline phosphatase (ALP), and lactate dehydrogenase (LDH) levels were all within normal range. Data were presented as the mean ± SD, n=1 experiment, n=6 mice per group.

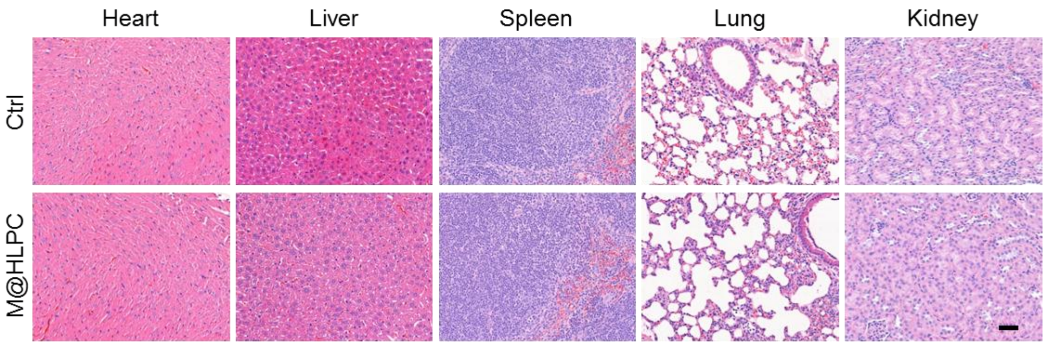

Supplemenetary Fig. 23 H&E staining of heart, liver, spleen, lung, and kidney slices after treatment with M@HLPC in U251-luc tumor-bearing mice (Ctrl: healthy mice without tumor and M@HLPC treatment). M@HLPC treatment showed no abnormality to above organs. Scale bar: 50 μm. Images were representative of three independent mice.

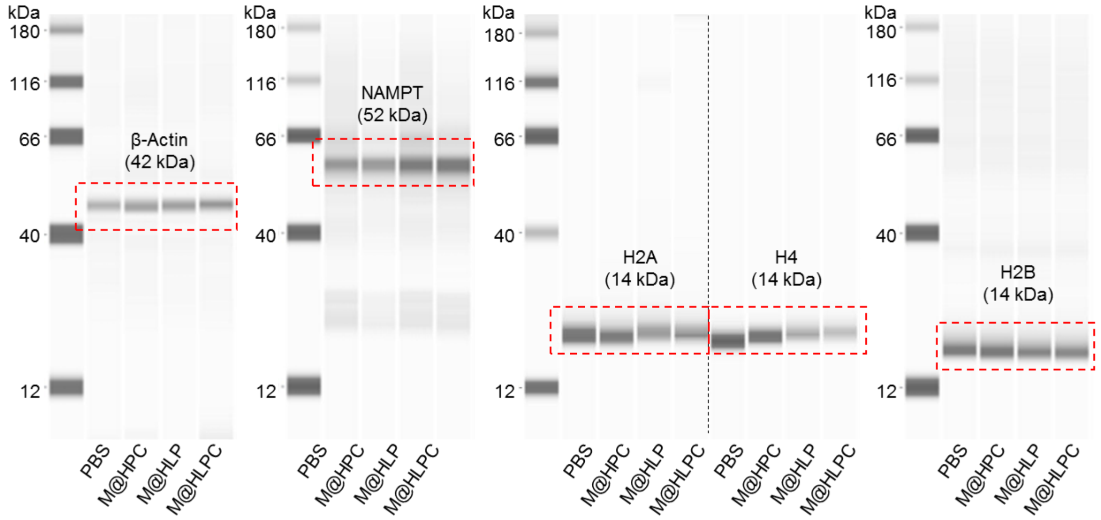

Supplemenetary Fig. 24 The full scans of Fig. 7g. Intracellular NAMPT and histones (H2A, H2B, and H4) levels in tumor tissues analyzed using ProteinSimple Wes™ Capillary Western Blot analyzer. Activation of NAMPT and repression of histone were observed in the tumors of mice treated with M@HPC and M@HLPC (LOX containing groups). These data were derived from the same batch experiment.

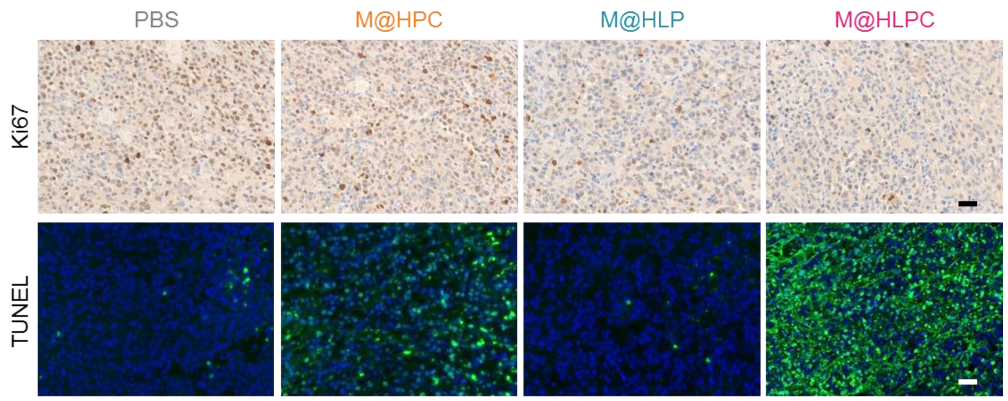

Supplemenetary Fig. 25 Representative cell proliferation (indicated by Ki67) and apoptosis (indicated by TUNEL) analysis of U251-luc tumor tissue. The results showed the highest degree of cells proliferation termination and apoptosis for the M@HLPC group. Scale bar: 50 μm. Images were representative of three independent mice.

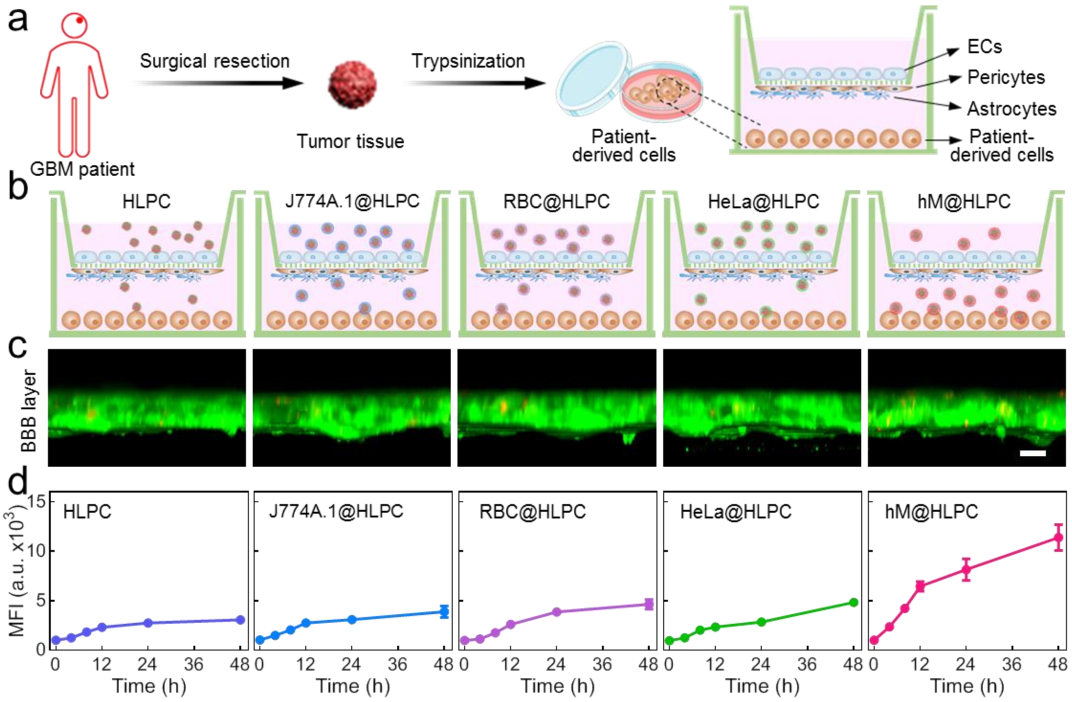

Supplemenetary Fig. 26 In vitro BBB penetration ability of hM@HLPC. (a) Schematic illustration of the experimental design for GBM patient-derived cells preparation and in vitro BBB model (Transwell™) construction. (b) Schematic illustration of the BBB model for evaluating the potential BBB penetration ability of the bare HLPC and those coated with diverse M. (c) Representative images of the BBB layer showed the penetration ability of various NPs. Red: NPs; green: DIO-labeled BBB layer cell membrane. Scale bar: 25 μm. (d) Quantification analysis of time-dependent internalization of NPs by GBM patient-derived cells. Data in d were presented as the mean ± SD, n = 3 independent samples. The experiments in c were repeated independently three times with similar results.

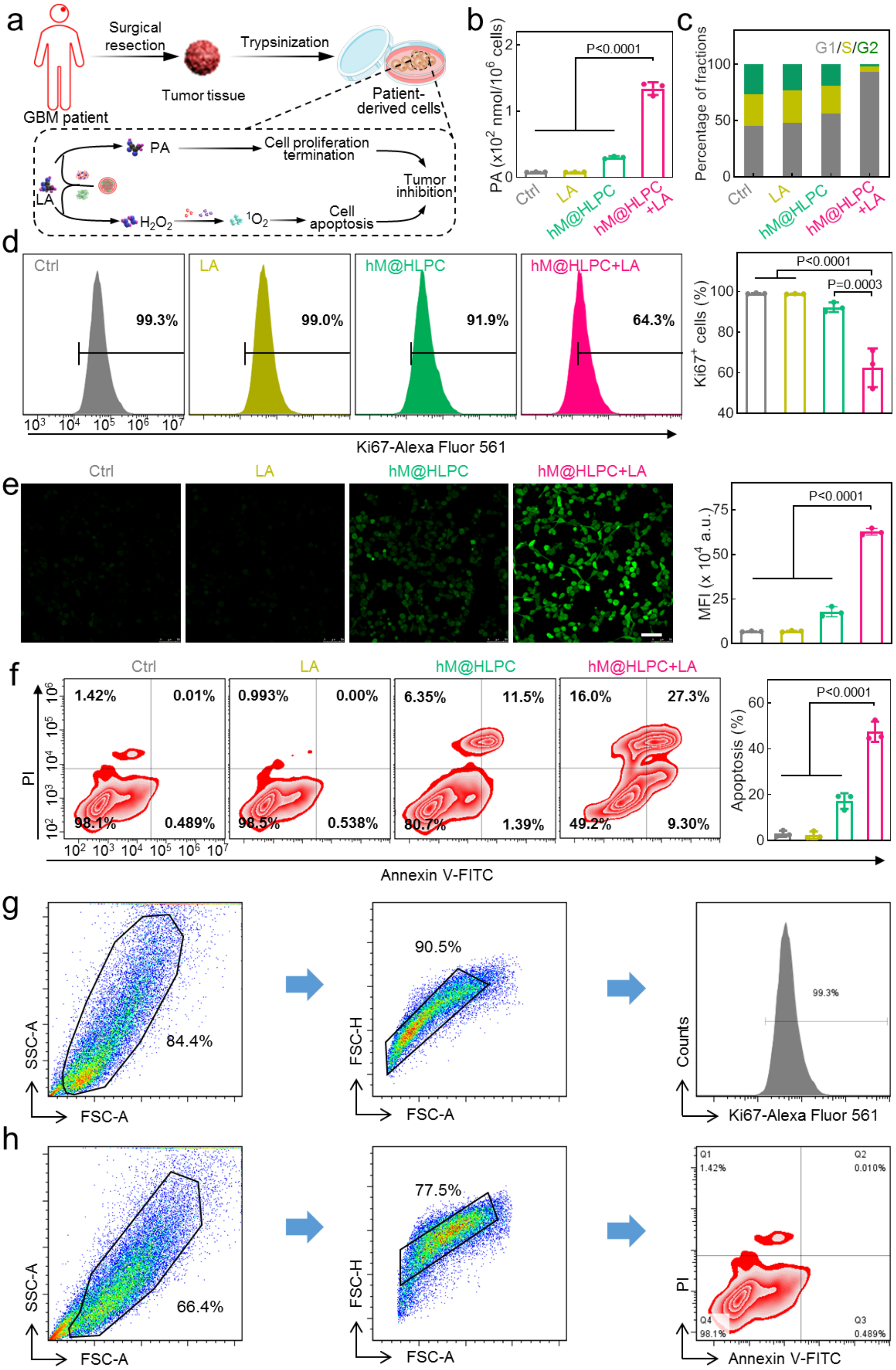

Supplemenetary Fig. 27 In vitro evaluation of the synergistic therapeutic effects of hM@HLPC against GBM patient-derived cells. (a) Schematic illustration of patient-derived cells preparation for evaluating synergistic antitumor mechanism of

hM@HLPC. (b) Quantitative analysis of PA concentration in patient-derived cells receiving the following different treatments: PBS, LA, hM@HLPC, or hM@HLPC+LA. (c) Cell cycle progression with different treatments, determined by flow cytometry. G1, S, and G2 represented G1 phase, S phase, and G2 phase of cell division cycle, respectively. All cells were synchronized to the G1 phase with 1.5 mM HU before subjecting to the indicated treatments. (d) Flow cytometry and corresponding quantitative analysis of patient-derived cell proliferation by Ki67 staining. (e) Representative CLSM images and corresponding quantitative analysis of DCFH-DA for the generation of $^1O_2$ in patient-derived cells receiving the indicated treatments. Scale bar: 50 μm. (f) Flow cytometry and corresponding quantitative analysis of the extent of patient-derived cell apoptosis by Annexin V/PI staining. (g) Flow cytometry gating strategy for d. (h) Flow cytometry gating strategy for f. Quantitative data in b and d-f were presented as the mean ± SD, n = 3 independent samples. The experiments in e were repeated independently three times with similar results. P values were calculated by using one-way ANOVA.

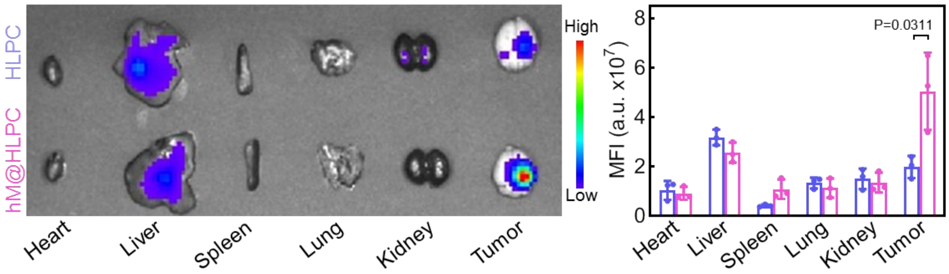

Supplemenetary Fig. 28 Representative ex vivo fluorescent images and corresponding quantitative fluorescence analysis of glioma tumors and major organs dissected from PDX models at 24 h after i.v. injection with HLPC or hM@HLPC. The results showed that coating HLPC with hM from human tumor tissue augments their PDX tumor-targeting abilities. Quantitative data were presented as the mean ± SD, n=1 experiment, n=3 mice per group. Images were representative of three independent mice. P value was calculated by using two-tailed unpaired Student's *t*-test.

Supplemenetary Table 1. List of cell membrane proteins selected basing on their function. This list includes typical proteins involved in cell interaction and cell adhesion that were enriched in the U251 cell rather than healthy astrocyte membrane sample.

Selected proteins	Molecular function	Ref.
CD44 antigen	Cell-cell interaction	1, 2
Zyxin	Focal adhesion	3
Cadherin-2	Cell-cell interaction	4, 5
Neuroplastin	Cell-cell interaction	6
Catenin beta-1	Cell adhesion regulation	7
Catenin delta-1	Cell adhesion regulation	8
Caveolae-associated protein 3	Regulates caveola formation and trafficking	9
Tight junction protein ZO-1	Plays a role in tight junction and adherens junction	10
Ras-related protein (Rab-22A)	Intracellular membrane trafficking	11
Vezatin	Cell-cell interaction	12
Neural cell adhesion molecule L1	Cell adhesion regulation	13
Ninjurin-1	Cell adhesion regulation	14
Transforming growth factor-beta-induced protein	Cell adhesion regulation	15
Collagen alpha-1(V) chain	Cell adhesion regulation	16
Nexilin	Cell adhesion regulation	17
CCN family member 1	Cell adhesion regulation	18
Insulin-like growth factor-binding protein 7	Cell adhesion regulation	19
Claudin-11	Cell adhesion regulation	20
Macrosialin	Cell adhesion regulation	21
CD99 antigen	Cell adhesion regulation	22
Integrin α4	Cell adhesion molecule binding	23
Integrin β1	Cell adhesion molecule binding	23

Supplemenetary Table 2. List of antibodies used in this study

Antibodies	Source	Identifier	Reactivity	Dilution
MCT4	Abcam<br>proteintech®	ab244385<br>22787-1-AP	human<br>human, mouse, rat	1:200 (IHC)<br>1:200 (IHC)
LDHA	proteintech®	66287-1-Ig	human, mouse, rat	1:200 (IHC)
Ki67	Abcam	ab15580	human, mouse	1:500 (IHC)<br>1:500 (FCM)
MEF2C	Abcam	ab211493	human, mouse, rat	1:500 (ICC)<br>1:200 (WB)
NAMPT	proteintech®	11776-1-AP	human, mouse, rat	1:500 (ICC)<br>1:100 (WB)
H2A	proteintech®	10445-1-AP	human, mouse	1:25 (WB)
H2B	Cell Signaling	12364	human, mouse, rat, monkey	1:25 (WB)
H4	Abcam	ab10158	human, mouse, rat	1:10 (WB)
β-Action	Abcam	ab6276	human, mouse, rat, cow, dog	1:200 (WB)
VCAM1	Abcam	ab134047	human, mouse, rat	1:200 (FCM)
CD44	Abcam	ab264539	human	1:100 (FCM)
Cadherin-2	Abcam	ab245117	human, mouse, rat	1:48.1 (FCM)
Zyxin	Abcam	ab109316	human, mouse, rat	1:93.7 (FCM)

ICC: Immunocytochemistry; IHC: Immunohistochemistry; WB: Western blotting; FCM: Flow cytometry.

Supplemenetary Table 3. List of siRNA used in this study

Gene name	Sequence
Scrambled siRNA	5’-UUCUCCGAACGUGUCACGUTT-3’<br>5’-UUGAUGUCUAUGGCCUGGCTT-3’
MEF2C siRNA (#1)	5’-CGUGGAGACGUUGAGAAAGAATT-3’<br>5’-UUCUUUCUCAACGUCUCCACGTT-3’
MEF2C siRNA (#2)	5’-GCACUCAUUUAUCUCAGAGUUTT-3’<br>5’-AACUCUGAGAUAAAUGAGUGCTT-3’
NAMPT siRNA (#1)	5’-GUAACUUAGAUGGUCUGGAAUTT-3’<br>5’-AUUCCAGACCAUCUAAGUUACTT-3’
NAMPT siRNA (#2)	5’-GGUAAGAAGUUUCCUGUUATT-3’<br>5’-UAACAGGAAACUUCUUACCTT-3’

Supplementary References

1 Lyu, C. L. et al. Engineering magnetosomes with chimeric membrane and hyaluronidase for efficient delivery of HIF-1 siRNA into deep hypoxic tumors. Chem. Eng. J. **398**, 125453 (2020).

2 Sun, H. P. et al. Cancer-Cell-Biomimetic Nanoparticles for Targeted Therapy of Homotypic Tumors. Adv. Mater. **28**, 9581-9588 (2016).

3 Fraley, S. I. et al. A distinctive role for focal adhesion proteins in three-dimensional cell motility. Nat. Cell Biol. **12**, 598-U169 (2010).

4 Jeanes, A., Gottardi,C. J., Yap, A.S. Cadherins and cancer: how does cadherin dysfunction promote tumor progression? Oncogene **27**, 6920-6929 (2008).

5 Perez, T. D., Nelson, W.J. Cadherin Adhesion: Mechanisms and Molecular Interactions. Cell Adhes. **165**, 3-21 (2004).

6 Bhattacharya, S. et al. Genetically Induced Retrograde Amnesia of Associative Memories After Neuroplastin Ablation. Biol. Psychiatry **81**, 124-135 (2017).

7 Lan, J. et al. Role of glycosyltransferase PomGnT1 in glioblastoma progression. Neuro-Oncology **17**, 211-222 (2015).

8 Fearnley, G. W. et al. The homophilic receptor PTPRK selectively dephosphorylates multiple junctional regulators to promote cell-cell adhesion. Elife **8**, e44597 (2019).

9 Park, P. J. & Kim, S. T. Caveolae-Associated Protein 3 (Cavin-3) Influences Adipogenesis via TACE-Mediated Pref-1 Shedding. Int. J. Mol. Sci. **21**, 5000 (2020).

10 Van Itallie, C. M. et al. The N and C Termini of ZO-1 Are Surrounded by Distinct Proteins and Functional Protein Networks. J. Biol. Chem. **288**, 13775-13788 (2013).

11 Kelly, M. R. et al. Combined Proteomic and Genetic Interaction Mapping Reveals New RAS Effector Pathways and Susceptibilities. Cancer Discovery **10**, 1950-1967 (2020).

12 Kussel-Andermann, P. et al. Vezatin, a novel transmembrane protein, bridges myosin VIIA to the cadherin-catenins complex. Embo J. **19**, 6020-6029 (2000).

13 Colombo, F. & Meldolesi, J. L1-CAM and N-CAM: From Adhesion Proteins to Pharmacological Targets. Trends Pharmacol. Sci. **36**, 769-781 (2015).

14 Horiuchi, K. et al. Pericyte-specific deletion of ninjurin-1 induces fragile vasa vasorum formation and enhances intimal hyperplasia of injured vasculature. Am. J. Physiol-Heart. C. **320**, H2438-H2447 (2021).

15 Ween, M. P., Oehler, M. K. & Ricciardelli, C. Transforming growth Factor-Beta-Induced Protein (TGFBI)/(betaig-H3): a matrix protein with dual functions in ovarian cancer. Int. J. Mol. Sci. **13**, 10461-10477 (2012).

16 Hatai, M., Hashi, H., Kato, I. & Yaoi, Y. Inhibition of Cell-Adhesion by Proteolytic Fragments of Type-V Collagen. Cell Struct. Funct. **18**, 53-60 (1993).

17 Ohtsuka, T. et al. Nexilin: A novel actin filament-binding protein localized at cell-matrix adherens junction. J. Cell Biol. **143**, 1227-1238 (1998).

18 Kubota, S. & Takigawa, M. CCN family proteins and angiogenesis: from embryo to adulthood. Angiogenesis **10**, 1-11 (2007).

19 Jiang, W. et al. Expression and function of insulin-like growth factor binding protein 7 (IGFBP-7/ IGFBP-rP1) in glioma cell. Cancer Res. **66**, 3296 (2006).

20 Denninger, A. R. et al. Claudin-11 Tight Junctions in Myelin Are a Barrier to Diffusion and Lack Strong Adhesive Properties. Biophys. J. **109**, 1387-1397 (2015).

21 Sawka-Verhelle, D. et al. PE-1/METS, an antiproliferative Ets repressor factor, is induced by CREB-1/CREM-1 during macrophage differentiation. J. Biol. Chem. **279**, 17772-17784 (2004).

22 Schenkel, A. R., Mamdouh, Z., Chen, X., Liebman, R. M. & Muller, W. A. CD99 plays a major role in the migration of monocytes through endothelial junctions. Nat. Immunol. **3**, 143-150 (2002)

23 Garmy-Susini, B. et al. Integrin α4β1-VCAM-1-mediated adhesion between endothelial and mural cells is required for blood vessel maturation. J. Clin. Invest. **115**, 1542–1551 (2005).
