# Supplementary material for: Engineered biomimetic nanoparticles achieve targeted delivery and efficient metabolism-based synergistic therapy against glioblastoma
Source: Nat Commun. 2022 Jul 21;13:4214. doi: 10.1038/s41467-022-31799-y (PMC9304377; doi:10.1038/s41467-022-31799-y)
Supplement: Supplementary file 2 — Reporting Summary [file 41467_2022_31799_MOESM2_ESM.pdf]

nature portfolio

Corresponding author(s): Guanghui Ma, Wei Wei, Weiping Li, Hui Tan

Last updated by author(s): Jun 20, 2022

Reporting Summary

Nature Portfolio wishes to improve the reproducibility of the work that we publish. This form provides structure for consistency and transparency in reporting. For further information on Nature Portfolio policies, see our Editorial Policies and the Editorial Policy Checklist.

Statistics

For all statistical analyses, confirm that the following items are present in the figure legend, table legend, main text, or Methods section.

n/a	Confirmed	
☐	☒	The exact sample size (n) for each experimental group/condition, given as a discrete number and unit of measurement
☐	☒	A statement on whether measurements were taken from distinct samples or whether the same sample was measured repeatedly
☐	☒	The statistical test(s) used AND whether they are one- or two-sided<br>*Only common tests should be described solely by name; describe more complex techniques in the Methods section.*
☒	☐	A description of all covariates tested
☐	☒	A description of any assumptions or corrections, such as tests of normality and adjustment for multiple comparisons
☐	☒	A full description of the statistical parameters including central tendency (e.g. means) or other basic estimates (e.g. regression coefficient) AND variation (e.g. standard deviation) or associated estimates of uncertainty (e.g. confidence intervals)
☐	☒	For null hypothesis testing, the test statistic (e.g. F, t, r) with confidence intervals, effect sizes, degrees of freedom and P value noted<br>*Give P values as exact values whenever suitable.*
☒	☐	For Bayesian analysis, information on the choice of priors and Markov chain Monte Carlo settings
☒	☐	For hierarchical and complex designs, identification of the appropriate level for tests and full reporting of outcomes
☒	☐	Estimates of effect sizes (e.g. Cohen's d, Pearson's r), indicating how they were calculated

Our web collection on statistics for biologists contains articles on many of the points above.

Software and code

Policy information about availability of computer code

Data collection	The images of immunohistochemistry (IHC) were visualized by using an automatic multispectral imaging system with associated software (Vectra II, PerkinElmer, version 2.0.7.1). Transmission electron microscope (TEM) images were recorded on JEOL JEM-1400 with associated software (version 1.7.18.2349). The size and zeta potential of HLPC and M@HLPC were measured by using a nanoparticle tracking analysis (NTA, Particle Metrix, Germany, version 8.05.04). The colloid stability of M@HLPC was measured via a ZetaSizer (NANO ZS, Malver, version 7.12). UV/vis absorption and fluorescence spectra were obtained with an automatic microplate reader (Tecan Infinite M200, version 1.6.19.2). Fe concentration in free Hb or HLPC was measured by inductively coupled plasma optical emission spectrometer (ICP-OES, iCAP 6300, version 2.8.0.89). The interaction between each sub-component in HLPC was measured by microscale thermophoresis (MST, NT.115, Nanotemper, Germany, version 2.3). Fluorescent images were collected using confocal laser scanning microscopy (CLSM, NIKON, A1, version 5.20.00). Flow cytometry data was acquired with Backman CytoFLEX LX Flow Cytometer with associated software (version 2.3.1.22). Two-photon microscopy imaging was performed with Leica TCS SP8 (Lecia, Germany, version 3.0). Magnetic resonance imaging (MRI) images were obtained on BioSpec 70/20 USR (Bruker, version 6.01). The images of western blotting were obtained using a ProteinSimple WesTM Capillary Western Blot analyzer (PS-MK15, ProteinSimple, version 6.0.0). The biodistribution of nanoparticles were observed by using IVIS imaging system (PerkinElmer, USA, version 4.5.5). The mass spectrometry was conducted using Q Exactive™ Hybrid Quadrupole-Orbitrap™ Mass Spectrometer with associated software (Thermo Proteome Discoverer, version 2.5.0.400)
Data analysis	All data were presented as mean ± SD. Statistical analysis was performed with GraphPad Prism 8.0.1 software by two-tailed unpaired Student’s t-tests, Log-rank test, or One-way ANOVA.

For manuscripts utilizing custom algorithms or software that are central to the research but not yet described in published literature, software must be made available to editors and reviewers. We strongly encourage code deposition in a community repository (e.g. GitHub). See the Nature Portfolio guidelines for submitting code & software for further information.

Data

Policy information about availability of data

All manuscripts must include a data availability statement. This statement should provide the following information, where applicable:

- Accession codes, unique identifiers, or web links for publicly available datasets
- A description of any restrictions on data availability
- For clinical datasets or third party data, please ensure that the statement adheres to our policy

The main data supporting the results in this study are available within the paper and its Supplementary Information. Source data are provided with this paper. Source data for the figures in the main text are available at Figshare (https://doi.org/10.6084/m9.figshare.19825075.v1). Source data for the figures in the Supplementary Information are available at Figshare (https://doi.org/10.6084/m9.figshare.19825096.v1). The RNA-seq has been deposited to NCBI under accession codes PRJNA747130 (https://www.ncbi.nlm.nih.gov/sra/PRJNA747130). The mass spectrometry proteomics data has been deposited to the ProteomeXchange Consortium via the iProX partner repository with the dataset identifier PXD030760 (http://proteomecentral.proteomexchange.org/cgi/GetDataset?ID=PXD030760). The dataset of LGG and HGG from TCGA were freely assessed on cBioportal (URL: http://www.cbioportal.org/).

Field-specific reporting

Please select the one below that is the best fit for your research. If you are not sure, read the appropriate sections before making your selection.

☒ Life sciences ☐ Behavioural & social sciences ☐ Ecological, evolutionary & environmental sciences

For a reference copy of the document with all sections, see nature.com/documents/nr-reporting-summary-flat.pdf

Life sciences study design

All studies must disclose on these points even when the disclosure is negative.

Sample size	Sample size was determined according to pilot studies as well as on the basis of previous experimental experiences. For in vitro and ex vivo studies, the sample size in each group was 3 independent samples. For the studies of tumor therapeutic outcomes, the sample size in each group was 6 independent mice.
Data exclusions	No data were excluded.
Replication	All attempts to replicate results were successful. In in vitro studies, experiments were biologically replicated three times and obtained similar results. For all murine experiments, we report pooled results from multiple experiments with similar results or the data shown correspond to one representative experiment of at least 3 biological replicates.
Randomization	For the in vitro experiments, samples were randomly allocated into experimental groups. For the in vivo studies, animals were randomly grouped.
Blinding	During data analysis for the experiments in this manuscript, we were blinded to group allocation and randomly allocated into experimental groups.

Reporting for specific materials, systems and methods

We require information from authors about some types of materials, experimental systems and methods used in many studies. Here, indicate whether each material, system or method listed is relevant to your study. If you are not sure if a list item applies to your research, read the appropriate section before selecting a response.

Materials & experimental systems

n/a	Involved in the study	
☐	☒	Antibodies
☐	☒	Eukaryotic cell lines
☒	☐	Palaeontology and archaeology
☐	☒	Animals and other organisms
☐	☒	Human research participants
☒	☐	Clinical data
☒	☐	Dual use research of concern

Methods

n/a	Involved in the study	
☒	☐	ChIP-seq
☐	☒	Flow cytometry
☒	☐	MRI-based neuroimaging

Antibodies

Antibodies used	Immunocytochemistry:<br>anti-MEF2C (abcam, ab211493, human, mouse, rat, 1:500 dilution)

anti-NAMPT (proteintech, 11776-1-AP, human, mouse, rat, 1:500 dilution);

Immunohistochemistry:
anti-MCT4 (abcam, ab244385, human, 1:200 dilution; proteintech, 22787-1-AP, human, mouse, rat, 1:200 dilution)
anti-Lactate Dehydrogenase A (proteintech, 66287-1-Ig, human, mouse, rat, 1:200 dilution)
anti-Ki67 (abcam, ab15580, human, mouse, 1:500 dilution)

Western blotting:
anti-MEF2C (abcam, ab211493, human, mouse, rat, 1:200 dilution);
anti-NAMPT (proteintech, 11776-1-AP, human, mouse, rat, 1:100 dilution);
anti-Histone H2A (proteintech, 10445-1-AP, human, mouse, 1:25 dilution);
anti-Histone H2B (Cell Signaling, 12364, human, mouse, rat, monkey, 1:25 dilution);
anti-Histone H4 (abcam, ab10158, human, mouse, rat, 1:10 dilution);
anti-β-Action (abcam, ab6276, human, mouse, rat, cow, dog, 1:200 dilution);

Flow cytometry:
anti-VCAM1 (abcam, ab134047, human, mouse, rat, 1:200 dilution);
anti-Ki67 (abcam, ab279653, human, mouse, rat, 1:500 dilution)

Blocking assay.
anti-CD44 (abcam, ab264539, human, 1:100 dilution);
anti-Cadherin-2 (abcam, ab245117, human, mouse, rat, 1:48.1 dilution);
anti-Zyxin (abcam, ab109316, human, mouse, rat, 1:93.7dilution).

Validation

We relied on publications and on validation sources cited by manufacturer. Links for each antibody are given below:

anti-MEF2C (abcam, ab211493, human, mouse, rat)
https://www.abcam.cn/mef2c-antibody-epr19089-202-chip-grade-ab211493.html
anti-NAMPT (proteintech, 11776-1-AP, human, mouse, rat);
https://www.ptgcn.com/products/NAMPT-Antibody-11776-1-AP.htm
anti-MCT4 (abcam, ab244385, human)
https://www.abcam.cn/slc16a3mct-4-antibody-ab244385.html
anti-MCT4 (proteintech, 22787-1-AP, human, mouse, rat)
https://www.ptgcn.com/products/SLC16A3-Antibody-22787-1-AP.htm
anti-Lactate Dehydrogenase A (proteintech, 66287-1-Ig, human, mouse, rat)
https://www.ptgcn.com/products/LDHA-Antibody-66287-1-Ig.htm
anti-Ki67 (abcam, ab15580, human, mouse)
https://www.abcam.cn/ki67-antibody-ab15580.html
anti-Histone H2A (proteintech, 10445-1-AP, human, mouse);
https://www.ptgcn.com/products/HIST3H2A-Antibody-10445-1-AP.htm
anti-Histone H2B (Cell Signaling, 12364, human, mouse, rat, monkey);
https://www.cellsignal.cn/products/primary-antibodies/histone-h2b-d2h6-rabbit-mab/12364?site-search
type=Products&N=4294956287&Ntt=12364&fromPage=plp&_requestid=1937742
anti-Histone H4 (abcam, ab10158, human, mouse, rat);
https://www.abcam.cn/histone-h4-antibody-chip-grade-ab10158.html
anti-β-Action (abcam, ab6276, human, mouse, rat, cow, dog);
https://www.abcam.cn/beta-actin-antibody-ac-15-ab6276.html
anti-VCAM1 (abcam, ab134047, human, mouse, rat);
https://www.abcam.cn/vcam1-antibody-epr5047-ab134047.html
anti-Ki67 (abcam, ab279653, human, mouse, rat);
https://www.abcam.cn/ki67-antibody-b56-ab279653.html
anti-CD44 (abcam, ab264539, human);
https://www.abcam.cn/cd44-antibody-c44mab-5-ab264539.html
anti-Cadherin-2 (abcam, ab245117, human, mouse, rat);
https://www.abcam.cn/n-cadherin-antibody-epr22397-264-ab245117.html
anti-Zyxin (abcam, ab109316, human, mouse, rat).
https://www.abcam.cn/zyxin-antibody-epr4302-ab109316.html

Eukaryotic cell lines

Policy information about cell lines

Cell line source(s)

Human cervical carcinoma cell (HeLa, catalog no. CRM-CCL-2TM) and mouse mononuclear macrophages (J774A.1, catalog no. TIB-67TM) were obtained from the American Type Culture Collection (ATCC). The human brain endothelial cell (hCMEC/D3, catalog no. 337728) was purchased from BeNa Culture Collection. The luciferase-transfected glioblastoma cell lines (U251-luc, catalog no. CBP30207L and GL261-luc, catalog no. iCell-0059a) were maintained in our laboratory (Shenzhen Second People's Hospital, Shenzhen, China). Human astrocytes (catalog no. 1800) and brain vascular pericytes (catalog no. 1200) were purchased from Sciencell.

Authentication

We used widely used cell lines from commercial sources, and these cells were authenticated by STR profiling.

Mycoplasma contamination

All the cell lines were tested termly and they were negative for mycoplasma contamination.

Commonly misidentified lines (See ICLAC register)	In this study, no commonly misidentified cell lines were used.

Animals and other organisms

Policy information about studies involving animals; ARRIVE guidelines recommended for reporting animal research

Laboratory animals	Animals. Four-week-old female Balb/c nude (catalog no. 13001A) and C57BL/6 (catalog no. 11001A) mice were purchased from Vital River Laboratories (Beijing, China). Four-week-old female NOD.Cg-Prkdcscid Il2rgtm1Vst/Vst (NPG, catalog no. NPG-1) mice were purchased from Shanghai Model Organisms Center, Inc. All the model mice were raised in a standard environmentally controlled room (23 °C, with 55 ± 5% humidity and under a 12 h/12 h light/dark cycle).
Wild animals	This study did not involve wild animals.
Field-collected samples	This study did not involve samples collected from the field.
Ethics oversight	All animal experiments were performed in accordance with the Guide for the Care and Use of Laboratory Animals (China, GB/T 35892-2018). The animal protocol was approved by the Institutional Animal Care and Use Committees at the Institute of Process Engineering, Chinese Academy of Sciences (approval ID: IPEAECA2019701).

Note that full information on the approval of the study protocol must also be provided in the manuscript.

Human research participants

Policy information about studies involving human research participants

Population characteristics	Twenty-two cases of diffuse astrocytoma and oligodendroglioma (grade II), twenty-one cases of anaplastic astrocytoma and anaplastic oligodendroglioma (grade III) and twenty-three cases of glioblastoma multiforme (grade IV) were selected from Shenzhen Second People's Hospital. Resected GBM samples for PDX model construction were obtained from a GBM patient (age 46, female) in Shenzhen Second People's Hospital.
Recruitment	The tumor samples were obtained from glioma patients with informed consent and were reviewed by the pathologist and surgeon. Pathologist classified the type and grade of the tumors in accordance with the WHO histological grading of central nervous system tumors.
Ethics oversight	The study was approved by Ethics Committee of Shenzhen Second People's Hospital Clinical trials (20210507004-FS01).

Note that full information on the approval of the study protocol must also be provided in the manuscript.

Flow Cytometry

Plots

Confirm that:

☒ The axis labels state the marker and fluorochrome used (e.g. CD4-FITC).

☒ The axis scales are clearly visible. Include numbers along axes only for bottom left plot of group (a 'group' is an analysis of identical markers).

☒ All plots are contour plots with outliers or pseudocolor plots.

☒ A numerical value for number of cells or percentage (with statistics) is provided.

Methodology

Sample preparation	The treated cells were collected from glass bottom dishes, filtered into single cell suspensions, and stained using the anti-Ki67, an-VCAM1, Annexin V-FITC/PI, DHCF.
Instrument	The instrument for data collection is CytoFLEX LX (BECKMAN COULTER).
Software	FlowJo 7.6 and CytExpert 2.3.1.22
Cell population abundance	Post-sort purities were greater than 90%.
Gating strategy	Generally, cells was first gated on FSC-A/SSC-A. Singlet cells were usually gated using FSC-H and FSC-A.

☒ Tick this box to confirm that a figure exemplifying the gating strategy is provided in the Supplementary Information.
